# Orally Bioavailable SARS-CoV-2 Protease Inhibitors Bearing a Hydroxymethyl Ketone Warhead

**DOI:** 10.3390/v18080821

**Published:** 2026-07-26

**Authors:** N. G. R. Dayan Elshan, Karen C. Wolff, Frank O. Weiss, Sourav Ghorai, Gennadii Grabovyi, Katy Wilson, Laura Riva, Ashley K. Woods, James R. Pedroarena, Armen Nazarian, Yuyin Liu, Wrickban Mazumdar, Lirui Song, Neechi Okwor, Jacqueline Malvin, Malina A. Bakowski, Melanie G. Kirkpatrick, Amal Gebara-Lamb, Edward Huang, Vân T. B. Nguyen-Tran, Stuart M. Weston, Carly Dillen, Victor Chi, Shuangwei Li, Matthew B. Frieman, Sumit K. Chanda, Kyoung-Jin Lee, Case W. McNamara, Anil Kumar Gupta, Alireza Rahimi, Jian Jeffrey Chen, Sean B. Joseph, Peter G. Schultz, Arnab K. Chatterjee

**Affiliations:** 1 Calibr-Skaggs Institute for Innovative Medicines, The Scripps Research Institute, La Jolla, CA 92037, USAjpedroar@uci.edu (J.R.P.);; 2 Center for Pathogen Research, Department of Microbiology and Immunology, University of Maryland School of Medicine, Baltimore, MD 21201, USA; 3 Department of Chemistry, The Scripps Research Institute, La Jolla, CA 92037, USA

**Keywords:** HMK, hydroxymethyl ketone, protease inhibitors, 3CLpro, M^pro^, SARS-CoV-2, antivirals

## Abstract

The use of covalent warheads targeting the catalytic cysteine has been a cornerstone in the coronavirus main protease (M^pro^) inhibitor development. Various electrophilic motifs have been explored, including aldehydes, nitriles, ketoamides, and hydroxymethyl ketones (HMKs). Recent efforts have mostly centered around nitrile warheads, given the success of Nirmatrelvir in the clinic. However, it is essential to identify and develop alternative chemotypes with distinct chemical and pharmacological profiles to prepare for future pandemics. Among such alternatives, HMKs are of particular interest because they balance reduced intrinsic electrophilicity with an excellent selectivity profile. Nevertheless, early HMK-based compounds, such as the clinical-stage M^pro^ inhibitor PF-00835231, suffered from poor oral bioavailability and therefore required intravenous administration, with or without prodrug derivatization of the hydroxyl group. In this work, we describe our efforts to advance the HMK field by discovering **mCMX110**, a lead compound that exhibits superior potency, increased unbound exposure in vivo, and favorable oral bioavailability in preclinical studies.

## 1. Introduction

Severe acute respiratory syndrome coronavirus 2 (SARS-CoV-2) created significant disruption to everyday life and led to substantial mortality across the globe. Efforts by researchers over the last several years have resulted in vaccines and antiviral agents that have significantly reduced morbidity and mortality rates from SARS-CoV-2 [1,2]. Despite this, the fundamental risks of viral pandemics remain a global health concern among the scientific community due to continued viral evolution and the risk of emerging variants with altered transmissibility and therapeutic susceptibility [3]. The development of next-generation antivirals targeting conserved viral proteins is one of the most attractive ways to address this risk, ensuring preparedness against current and future coronaviruses.

The SARS-CoV-2 main protease (M^pro^, also termed 3CL^pro^) is an essential cysteine protease responsible for the proteolytic processing of viral polyproteins (pp1a and pp1ab) into functional nonstructural proteins required for replication and transcription [4]. M^pro^ cleaves at multiple conserved Leu-Gln↓ (Ser, Ala, Gly) motifs and lacks closely related human homologs, making it an attractive antiviral target with reduced risk of off-target toxicity [4]. Structural studies have revealed a catalytic Cys145–His41 dyad within a well-defined substrate binding pocket, enabling rational design of covalent and reversible covalent inhibitors [4,5,6].

**Figure 1:**
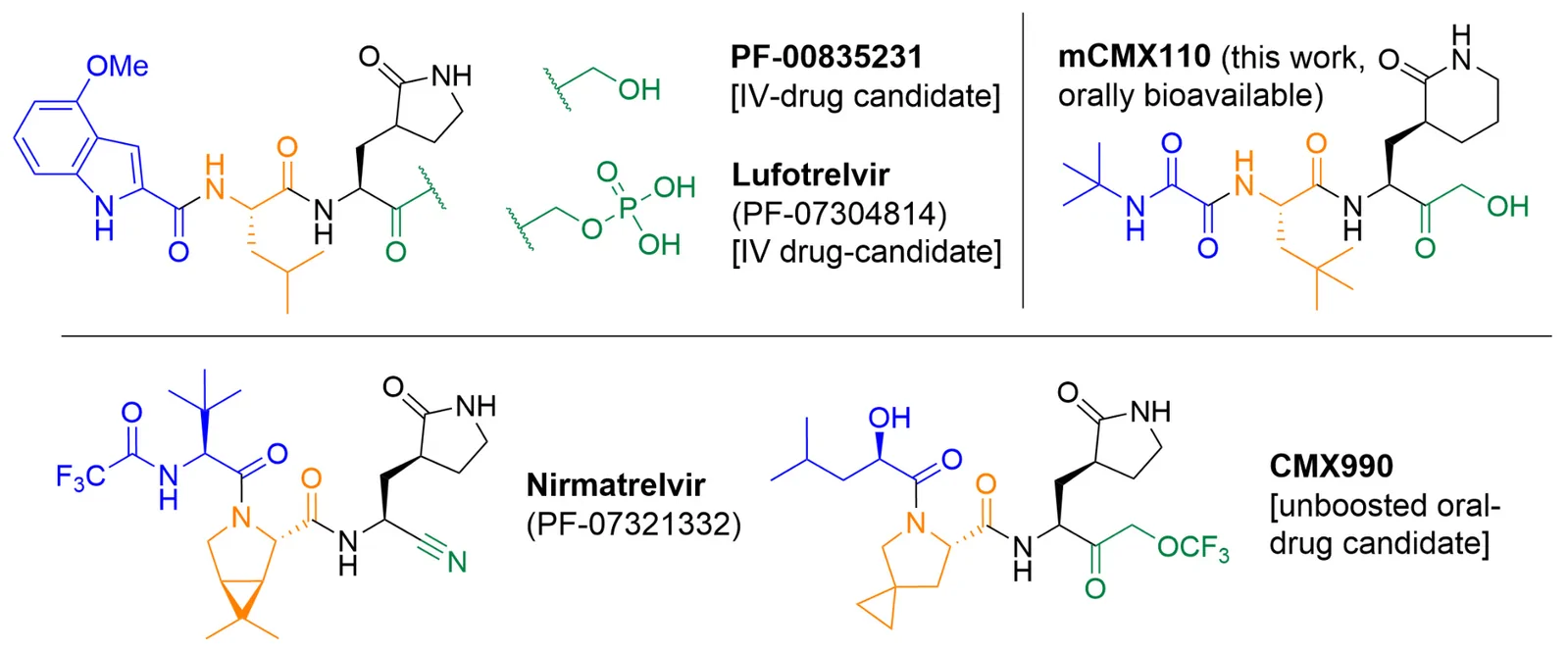
Representative known coronavirus protease inhibitors with various covalent warheads in comparison to the lead **mCMX110** from the current work. Generally, up to four distinct structural regions are found in all peptidomimetic CL^pro^ inhibitors: warhead (WH, green), P1 (amino acid 1, recognition element with a lactam side-chain, black), P2 (AA 2, orange), and P3 (a *N*-Cap, or a third AA, blue). The warheads can have distinct differences in electrophilicity that contribute substantially to the potency and pharmacokinetic performance of the compound. **mCMX110** (lead from this work) improves upon PF-00835231 by having superior oral bioavailability without a need for ritonavir-boosting or prodrug strategies.

Covalent warheads targeting the catalytic cysteine have been particularly successful in M^pro^ inhibitor development [7,8,9], particularly Nirmatrelvir [6]. Among these, electrophilic motifs including aldehydes, nitriles, ketoamides, and hydroxymethyl ketones (HMKs) form reversible covalent adducts with Cys145, stabilizing a tetrahedral hemithioacetal intermediate that mimics the transition state of peptide cleavage [10]. Hydroxymethyl ketones are of special interest because they balance reactivity and selectivity: they are sufficiently electrophilic to engage catalytic cysteines while typically exhibiting reduced nonspecific reactivity compared to other warheads [10]. HMK warheads have been previously validated versus cysteine proteases, supporting their suitability for targeting coronavirus proteases [10,11,12]. PF-00835231, the first-in-class SARS-CoV-1 M^pro^ inhibitor developed by Pfizer, is one of the key examples of an HMK-based drug candidate, but lacked the oral bioavailability to cover efficacious exposure against M^pro^. At the onset of the SARS-CoV-2 pandemic, PF-00835231 (Figure 1) was modified into a prodrug form (PF-07304814, Lufotrelvir, Figure 1) [12] and evaluated as an intravenous treatment in the clinic. Further development of these HMK-based M^pro^ inhibitors was abandoned with the discovery of orally bioavailable M^pro^ inhibitors such as Nirmatrelvir.

The recent clinical landscape of SARS-CoV-2 M^pro^ inhibitors is dominated by covalently associating warheads such as nitriles or emerging warheads, as seen in our recent clinical compound CMX990 [13]. These work well in many circumstances, but still have caveats regarding their overall pharmacokinetics, efficacy, and safety profile (e.g., Nirmatrelvir requires co-dosing with CYP-inhibitor ritonavir to boost exposure for target coverage). Given these shortcomings, the risks of resistance driving the mutations emerging in M^pro^ [14,15,16,17,18], and the overall need for preparedness against future pandemics, it is essential that we discover novel, structurally diverse chemotypes to expand the chemical space of M^pro^ inhibitors. To this end we disclose another chemical series in our SARS-CoV-2 M^pro^ inhibitor program, outlining the discovery of **mCMX110**, an HMK warhead bearing M^pro^ inhibitor that differentiates from PF-00835231 in having superior oral bioavailability without a need for ritonavir-boosting or prodrug strategies.

## 2. Materials and Methods

All reagents and solvents were purchased from commercial sources and used without further purification unless otherwise specified. Flash column chromatography was performed using silica gel (200–300 mesh). All reactions were monitored by TLC (pre-coated EMD silica gel 60 F254 TLC aluminum sheets and visualized with a UV lamp or appropriate stains) and/or LCMS (Waters Acquity UPLC system, 2 or 4 min run of a 10–90% mobile phase gradient of acetonitrile in water [+0.1% formic acid]). NMR spectra were obtained on AV400 or AV500 instruments (Bruker Corporation, MA, USA), and data was analyzed using the MestReNova NMR software (version 16.0, Mestrelab Research S. L., Santiago de Compostela, Spain). Chemical shifts (δ) are expressed in ppm and are internally referenced for ^1^H NMR (DMSO-*d*_6_ 2.50 ppm) and ^13^C NMR (CDCl_3_ 77.16 ppm, DMSO-*d*_6_ 39.52 ppm).

### 2.1. Chemistry

**Scheme 1:**
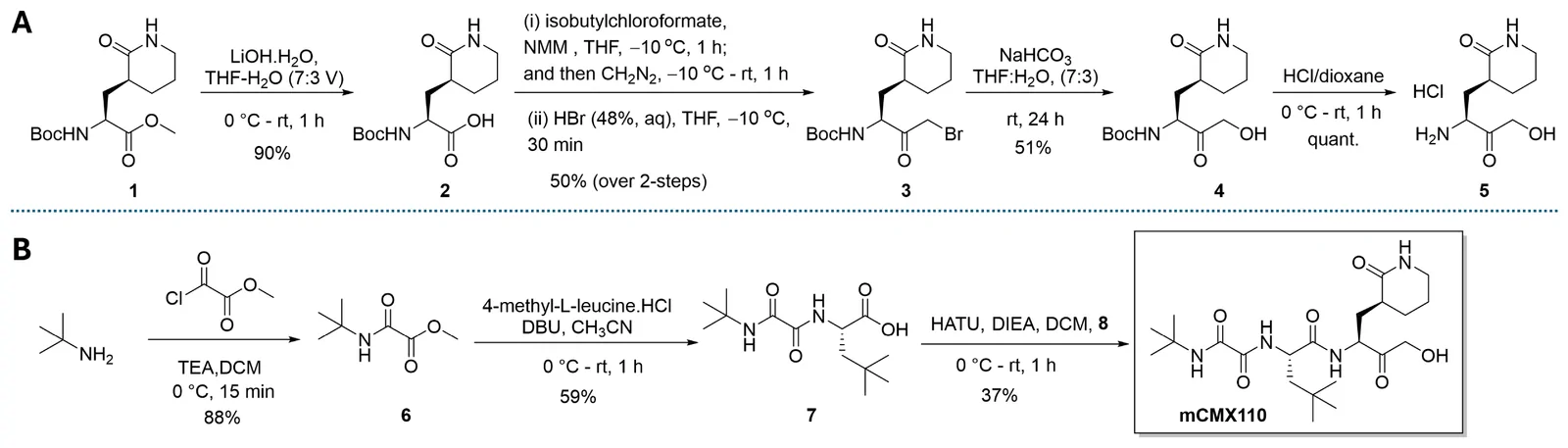
Synthesis of **mCMX110.** (**A**) Synthesis of HMK warhead containing P1-motif. (**B**) Final assembly of **mCMX110** (19% overall yield). This scheme is representative of the synthesis of all HMK compounds described in this text and was used to synthesize material at the gram scale.

All compounds with the hydroxymethylketone warhead were synthesized according to the representative procedure outlined for **mCMX110** (Scheme 1). All compounds described are >95% pure by HPLC and/or NMR analyses. As we have reported previously [13], some of these peptidomimetics demonstrate rotamer distributions in NMR.

((*S*)-2-((*tert*-butoxycarbonyl)amino)-3-((*S*)-2-oxopiperidin-3-yl)propanoic acid **(2):** To a stirred solution of methyl (*S*)-2-((*tert*-butoxycarbonyl)amino)-3-((*S*)-2-oxopyrrolidin-3-yl)propanoate **1** (10 g, 33.3 mmol, 1.0 eq) in tetrahydrofuran (70 mL), lithium hydroxide monohydrate (1.68 g, 40.0 mmol, 1.2 eq) in water (30 mL) was added at 0 °C and the mixture was stirred 1 h. After completion, the reaction mixture was cooled 0 °C, acidified with 2 N hydrochloric acid (pH~5) and extracted with ethyl acetate (3 × 300 mL). The combined organic layer was dried over anhydrous sodium sulphate and concentrated under reduced pressure to afford the free acid **2** (8.0 g, 30.0 mmol, 90%) as a pale yellow gum. TLC system: MeOH:DCM (1:9); *R_f_*: 0.1.

*tert*-butyl ((*S*)-4-bromo-3-oxo-1-((*S*)-2-oxopiperidin-3-yl)butan-2-yl)carbamate **(3):** To a stirred solution of **2** (8.0 g, 30.0 mmol, 1.0 eq) in tetrahydrofuran (100 mL), *N*-methylmorpholine (6.58 mL, 60.0 mmol, 2.0 eq) was added, then isobutyl chloroformate (6.13 mL, 44.9 mmol, 2.2 eq) was added at −10 °C and the reaction mixture was stirred at −10 °C for 1 h. After completion, the reaction mixture was filtered and washed with tetrahydrofuran (50 mL). Freshly prepared diazomethane in diethyl ether (prepared from 4.0 eq. of diazald) was added to the filtrate at −10 °C and the reaction mixture was stirred at room temperature for 1 h. After completion, the reaction mixture was diluted with water (100 mL) and extracted with ethyl acetate (3 × 300 mL). The combined organic layer was dried over anhydrous sodium sulphate and evaporated under reduced pressure to afford *tert*-butyl ((*S*)-4-diazo-3-oxo-1-((*S*)-2-oxopiperidin-3-yl)butan-2-yl)carbamate (9.8 g, crude) as a yellow liquid. TLC system: EtOAc:pet-ether (7:3); *R_f_*: 0.2.

To a stirred solution of the α-diazo derivative above (9.8 g, 31.6 mmol) in tetrahydrofuran (100 mL), 48% aqueous hydrobromic acid (6.4 mL, 37.94 mmol) was added dropwise at −10 °C and the mixture was stirred at −10 °C for 30 min. After completion, the reaction mixture was basified with saturated sodium bicarbonate and extracted with ethyl acetate (3 × 150 mL). The combined organic layer was dried over anhydrous sodium sulphate and concentrated under reduced pressure. The crude residue was then purified by column chromatography over silica gel (230–400 mesh) using 50–70% ethyl acetate in pet-ether as a gradient to afford the α-bromomethyl-ketone derivative **3** (5.4 g, 14.9 mmol, 50% over two steps) as a yellow liquid. TLC system: EtOAc:pet-ether (7:3); *R_f_*: 0.4.

*tert*-butyl ((*S*)-4-hydroxy-3-oxo-1-((*S*)-2-oxopiperidin-3-yl)butan-2-yl)carbamate (**4**): To the stirred solution of **3** (4.8 g, 14.9 mmol, 1.0 eq) in THF:H_2_O (35:15 mL), sodium bicarbonate (6.26 g, 74.6 mmol, 5.0 eq) was added and the reaction mixture was stirred at room temperature for 24 h. After completion, the reaction mixture was diluted with water (100 mL) and extracted with ethyl acetate (3 × 150 mL). The combined organic layer was dried over anhydrous sodium sulfate and evaporated under reduced pressure to yield crude product. The crude residue was then purified by silica gel (230–400 mesh) column chromatography using 5–7% MeOH in DCM as a gradient to afford α-hydroxymethyl-ketone **4** (2.3 g, 7.7 mmol, 51%) as an off-white solid. TLC system: EtOAc:pet-ether (7:3); *R_f_*: 0.5.

(*S*)-3-((*S*)-2-amino-4-hydroxy-3-oxobutyl)piperidin-2-one hydrochloride (**5**): A solution of **4** (3 g, 10.0 mmol, 1 eq) in DCM (45 mL) was treated with HCl/dioxane(4N) (10 mL, 40.0 mmol, 4.0 eq) for 15 min at 0 °C under nitrogen. The resulting mixture was stirred for 1 h at room temperature under nitrogen atmosphere. After the reaction was completed, the solvent was removed under vacuum to yield hydrochloride salt **5** (2.1 g, quant.) as a white solid. LCMS:(ES, *m/z*): [M + H]^+^ = 201.

Methyl 2-(*tert*-butylamino)-2-oxoacetate (**6**): To a stirred mixture of *tert*-butylamine (75 g, 1025 mmol, 1.0 eq) and TEA (311.30 g, 3076.3 mmol, 3 eq) in DCM (750 mL), methyl oxalochloridate (150.7 g, 1230.5 mmol, 1.2 eq) was added dropwise at 0 °C under nitrogen atmosphere. The resulting mixture was washed with 1N HCl (5 × 500 mL), brine (2 × 200 mL), dried over anhydrous Na_2_SO_4_. After filtration, the filtrate was concentrated under reduced pressure. The residue was purified by silica gel column chromatography, and eluted with n-hexane/EA (1:1) to afford methyl ester **6** (130 g, 902 mmol, 88%) as a yellow oil. LCMS:(ES, *m/z*): [M + H]^+^ = 160.

(2*S*)-2-[(*tert*-butylcarbamoyl) formamido]-4,4-dimethylpentanoic acid (**7**): To a stirred solution of 4-methyl-L-leucine (2.9 g, 20.0 mmol, 1.0 eq) and **6** (3.82 g, 24.0 mmol, 1.2 eq) in ACN (70 mL), DBU (7.60 g, 49.9 mmol, 2.5 eq) was added at 0 °C under nitrogen atmosphere. The resulting mixture was stirred for 1 h at room temperature under nitrogen atmosphere. After the reaction was completed, the mixture was diluted with EtOAc (200 mL), washed with 1N HCl (5 × 70 mL), brine (3 × 70 mL) and dried over anhydrous Na_2_SO_4_. After filtration, the filtrate was concentrated under reduced pressure. The residue was purified by silica gel column chromatography and eluted with PE/THF (1:1) to afford the acid **7** (3.2 g, 11.7 mmol, 59%) as a brown-yellow solid. LCMS:(ES, *m/z*): [M + H]^+^ = 273. ^1^H NMR (300 MHz, DMSO-*d*_6_): *δ* 8.75–8.72 (m, 1H), 7.82 (s, 1H), 4.27–4.21 (m,1H), 1.87–1.68 (m, 2H), 1.33 (s, 9H), 0.88 (s, 9H).

*N*-*tert*-butyl-*N*’-[(1S)-1-[[(2*S*)-4-hydroxy-3-oxo-1-[(3*S*)-2-oxopiperidin-3-yl] butan-2-yl] carbamoyl]-3,3-dimethylbutyl] ethanediamide (**mCMX110**): To a stirred solution of **7** (2.1 g, 7.7 mmol, 1.0 eq) and the hydrochloride **5** (2.19 g, 9.3 mmol, 1.2 eq) in DCM (70 mL), HATU (3.52 g, 9.2 mmol, 1.2 eq) and DIEA (2.49 g, 19.3 mmol, 2.5 eq) were added at 0 °C under nitrogen atmosphere. The resulting mixture was stirred for 1 h at room temperature under nitrogen atmosphere. After the reaction was completed, it was quenched with 10 mL water at 0 °C. The resulting mixture was washed with 1N HCl (5 × 70mL). The combined organic layers were washed with brine (50 mL), dried over anhydrous Na_2_SO_4_. After filtration, the filtrate was concentrated under reduced pressure. The residue was purified by silica gel column chromatography, and eluted with PE/THF (1:3) to afford **mCMX110** (1.3 g, 2.9 mmol, 37%) as a white solid. It was further purified by SFC to give 0.7 g, the final target, as a white amorphous solid.

SFC separation condition: Column: CHIRALPAK IH, 3 × 25 cm, 5 μm; mobile phase A: CO_2_, mobile phase B: IPA:ACN = 1:1; flow rate: 80 mL/min; gradient: isocratic 30% B; column temperature (°C): 35; back pressure (bar): 100; wavelength: 220 nm; RT1 (min): 2.63; RT2 (min): 5.27; sample solvent: IPA; injection volume: 5 mL; number of runs: 10).

ESI-MS *m/z* *calcd.* for C_22_H_39_N_4_O_6_^+^ [M + H]^+^ 455.3, found 455.3; ^1^H NMR (400 MHz, DMSO-*d*_6_): *δ* 8.61 (d, *J* = 9.0 Hz, 1H), 8.39 (d, *J* = 8.0 Hz, 1H), 7.84 (s, 1H), 7.46 (s, 1H), 5.09 (t, *J* = 6.0 Hz, 1H), 4.51–4.41 (m, 1H), 4.38–4.29 (m, 1H), 4.26–4.04 (m, 2H), 3.15–3.05 (m, 2H), 2.18–2.03 (m, 2H), 1.87–1.45 (m, 6H), 1.39–1.21 (m, 10H), 0.88 (s, 9H). ^13^C NMR (101 MHz, DMSO-*d*_6_) δ 210.03, 173.07, 172.34, 160.27, 159.16, 66.09, 53.08, 51.69, 51.28, 45.02, 41.67, 37.44, 31.79, 30.76, 29.92, 28.46, 25.99, 21.68.

### 2.2. In Vitro Biology

The following viral strains were obtained through BEI Resources, NIAID, NIH: Isolate HCOV-19 USA-WA1/2020 (NR-52281), isolate hCoV-19/USA/OR-OHSU-PHL00037/2021 (Alpha Variant, NR-55461), isolate HCOV-19/USA/PHC658/2021 (Delta variant, NR-55691), isolate HCOV-19/USA/MD-HP20874/2021 (Omicron variant, NR-56461), human coronavirus, OC43 (NR-52725), and human coronavirus, 229E (NR-52726). The SARS-CoV-2 assays (cell-based, enzymatic) were conducted as described previously [13,19].

SARS-CoV-1 and MERS-CoV assays were conducted in A549-ACE2 or A549-DPP4 cells respectively. Cells were seeded to black-welled 96 well plates (Costar 3603, Corning Inc., New York, NY, USA) one day prior to infection. The compounds to screen were diluted in a 1000× concentration DMSO stock dilution series, stored at −20 °C, then diluted in DMEM to a 2× final screening concentration on the day of infection. Infections were performed with a pre-determined concentration of virus to achieve between 30 and 50% infection. Virus was added to a volume of DMEM for a 1:1 mix with 2× compound containing media which were added to the cells for drug treatment and infection at the final concentration (10 μM compound [or as indicated on graphs]). Cells were incubated at 37 °C/5% CO_2_ for 24 h or 48 h for SARS-CoV and MERS-CoV respectively. Cells were fixed with 10% neutral buffered formalin for 24 h at 4 °C prior to immunofluorescence staining. Formalin was quenched with 50 mM NH_4_Cl, cells were permeabilized with 0.1% Triton-X100 diluted in 0.2% BSA in PBS (blocking buffer) for 10 min. Cells were then blocked with blocking buffer for 10 min and incubated with appropriate anti-N antibody (Sino Biologicals—Beijing, China, 40143-R004 or 40068-RP01, for SARS-CoV and MERS-CoV, respectively) for 1 h in blocking buffer. Cells were washed for 3 × 5 min in blocking buffer then incubated for 45 min with anti-rabbit AlexaFlour488 (A11008, Thermo Scientific, Waltham, MA, USA) diluted in blocking buffer. Cells were washed for a further 3 × 5 min with PBS and incubated with Hoechest 33342 (H3570, Thermo Scientific, MA, USA) diluted in PBS for 10 min. Cells were washed for a final 2 × 5 min with PBS and imaged with a Celigo high content imager (Nexcelom Bioscience LLC, Lawrence, MA, USA). Total cells were determined by Hoechst fluorescence and infected cells determined by AlexFluor488 signal. Uninfected wells were used to perform a background fluorescence subtraction, and percentage infection was determined for each well. Infection level was set relative to 0.1% DMSO control well average per plate and percentage inhibition was determined by 100% infection. These data were plotted in Prism (GraphPad) v11.0.2 on a log(inhibitor) vs. response–variable slope (four parameters) curve to calculate the IC_50_ values of each compound.

HCoV-OC43 was propagated in HCT-8 cells (ATCC CCL-244) and compound activity was assayed in a high-content imaging assay after a 48 h incubation using the mouse monoclonal antibody OC-43 strain clone 541-8F (MAB9012, MilliporeSigma, Burlington, MA, USA) and goat anti-mouse H+L conjugated Alexa 488 secondary (A11001, Thermo Fisher Scientific, MA, USA). HCoV-229E was propagated in MRC-5 pd25 cells (cat # 05081101-1VL, MilliporeSigma, MA, USA) and compound activity was determined by assessing cell viability at 120 h post-infection using CellTiter-Glo^®^ (No G7573, Promega corporation, Madison, WI, USA). The uninfected cytotoxicity counter screens were incubated in parallel with the antiviral assays and cell viability was assessed using CellTiter-Glo^®^.

### 2.3. Drug Metabolism and Pharmacokinetics (DMPK) Assays

The described in vitro ADME assays were conducted at Calibr-Skaggs (San Diego, CA, USA) or at WuXi AppTec (Shanghai, China) using the procedures previously described [13,19]. Pharmacokinetic studies [CD1-mouse, SD-rat, Syrian hamster, beagle dog] were conducted at Aragen (Hyderabad, India), Calibr (San Diego, CA, USA), WuXi (Wuxi, China), or Pharmaron Inc. (Beijing, China)

For all reported intravenous (IV) pharmacokinetic studies in this manuscript, three fasted animals per study group were administered the test article as a solution with 50% PEG 300, 10% ethanol, and 40% saline. For the oral PK studies, the same formulation was used for solutions, whereas for suspensions 0.5% methylcellulose (MC) or 0.5% sodium carboxymethyl cellulose (NaCMC) in water was used. Blood samples were collected per general protocol at 0.083, 0.5, 1, 3, 5, 8, and 24 h post-dosing for IV studies, and at 0.5, 1, 3, 5, 8, and 24 h post-dosing for PO studies.

The blood samples were centrifuged to obtain plasma, which was stored below −20 °C until analysis. Plasma concentrations were determined by liquid chromatography/tandem mass spectrometry (LC-MS/MS). The PK parameters were determined by non-compartmental methods using WinNonLin (v6.1 or higher version, Certara Inc., Princeton, NJ, USA).

## 3. Results

### 3.1. Syntheses of ***mCMX110*** Series Compounds Are Readily Feasible

The synthesis of this hydroxymethyl ketone compound series follows generally known synthetic chemistry methods described by us and others in the literature [6,11,13]. Representative synthesis of **mCMX110** (Scheme 1) was achieved with 19% yield for the three steps from *t*-butylamine.

Commercially available methyl *N*-Boc-protected glutamine ester **1** was hydrolyzed under mild conditions with LiOH in aqueous THF to afford carboxylic acid **2** in 90% yield (Scheme 1A). Conversion of acid **2** into the corresponding α-bromomethyl ketone **3** was accomplished through a classic diazoketone (Arndt–Eistert-type diazo transfer) [20]/hydrobromination sequence. Activation of the carboxylic acid with isobutyl chloroformate generated the mixed anhydride, which was trapped with diazomethane to furnish the corresponding diazoketone. Subsequent treatment with aqueous hydrobromic acid afforded α-bromomethyl ketone **3** in 50% overall yield over two steps. The bromide was then converted into hydroxymethyl ketone **4** via a bicarbonate-promoted hydrolysis reaction, providing the desired hydroxymethyl ketone in 51% yield. Subsequent Boc deprotection using hydrogen chloride in dioxane furnished the key amine hydrochloride **5** quantitatively. The P2/P3 fragment was synthesized convergently (Scheme 1B), first acylating *tert*-butylamine with methyl oxalyl chloride produced carbamate **6** in 88% yield. Coupling of **6** with 4-methyl-*L*-leucine hydrochloride under DBU-mediated aminolysis conditions generated oxamide **7** in 59% yield. Finally, fragments **5** and **7** were united through HATU-mediated amide bond formation in the presence of DIPEA to afford **mCMX110** in 37% isolated yield.

We used the currently disclosed sequence to make **mCMX110** in >20 g scales. Without the use of SFC at the final step, the major impurity is the epimerization-derived diastereomer impurity at P1. Based on our prior experiences with similar compounds, this process can be further optimized to avoid the need for SFC separation and improve the overall yield.

### 3.2. SAR Studies Establish ***mCMX110*** as Having the Best Balance Between Potency and Early ADME Properties

**Figure 2:**
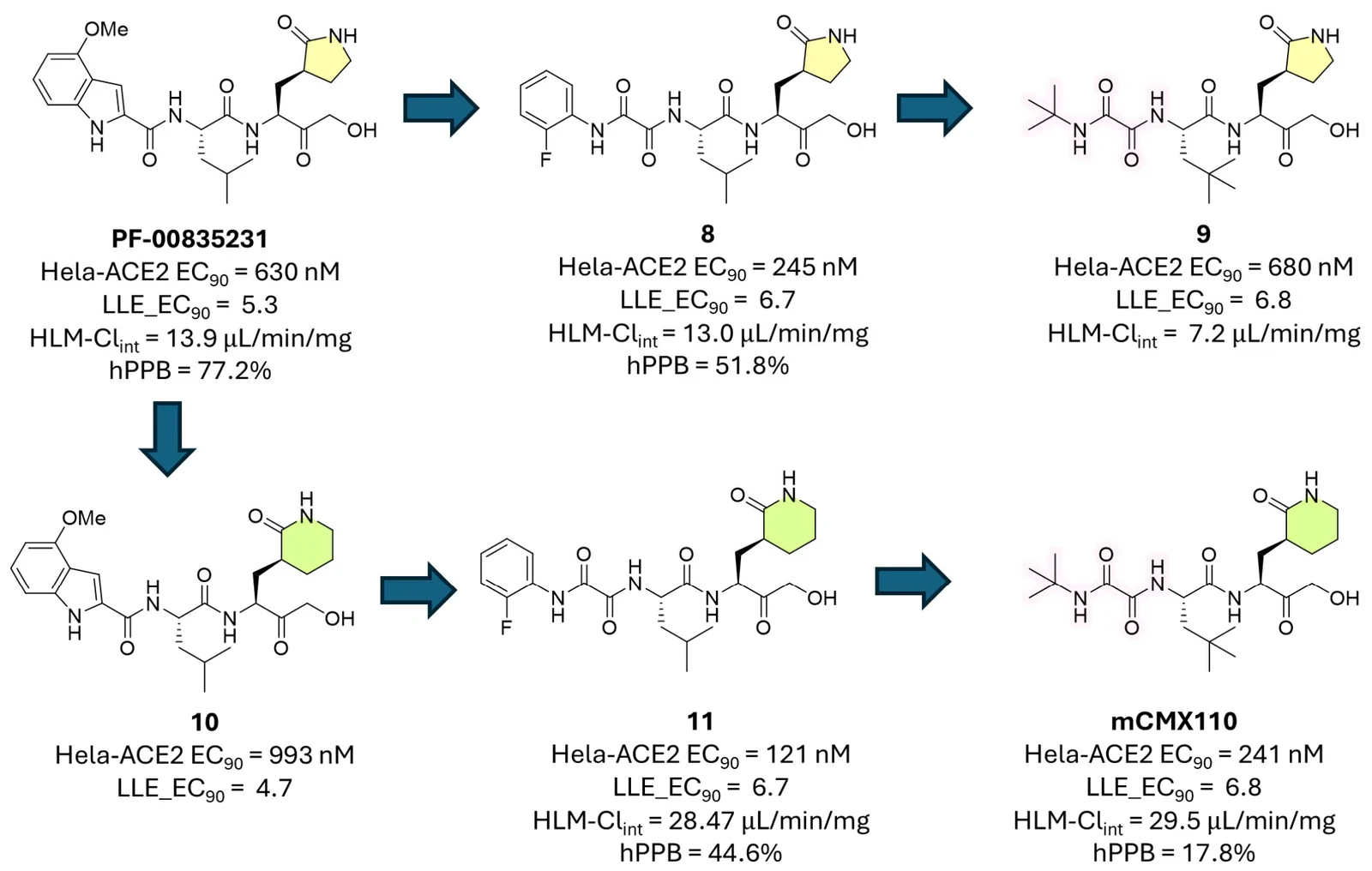
Representative SAR summary trajectory to early tool compound 8, and ultimately to lead **mCMX110.** SAR was carried out in parallel across all regions of the compounds, leading to a series of ~100 HMK compounds.

We synthesized ~100 analogs (representative SAR disclosed here) in this HMK series, where the key SAR improvements were found through oxamide P2-caps (Figure 2). Maintaining the γ-lactam (pyrrolidinone) P1 motif of PF-00835231 and bringing in the aryl-oxamide P2-cap in place of the methoxyindole led to an immediate improvement in potency (>2× improved) and lipophilic ligand efficiency (LLE, 1.4 units improvement), as represented by compound **8**.

Several aryl-oxamide-containing compounds demonstrated positive mini-Ames assay results, following which we decided to move away from the anilinic oxamides to minimize developmental risk. Among other modifications (Table 1), replacement of the para-fluorophenyl group of **8** with a t-butyl group resulted in analog **9** with 2× improvement in microsomal clearance, but compromised potency. To regain the potency, we aimed to replace the pyrrolidinone motif with a piperidinone P1 sidechain. Indeed, in most cases (e.g., **11** vs. **8**), the δ-lactam was superior to the γ-lactam counterpart in driving potency, albeit at a moderate cost for microsomal clearance. Eventually, our SAR exploration, presented in Table 1, led to the identification of lead compound **mCMX110**, which retained a potency of **8** without compromising LLE. Importantly, **mCMX110** had low plasma protein binding (human PPB almost 3× lower vs. **8**), which favorably increased the unbound fraction that is recognized as a key determinant of antiviral efficacy. They were still better in pairing with P2-prolines versus alternative P3-caps, such as the leucic acid P2-cap in **17** (CoV-2 EC_90_ > 10 µM).

Incorporation of other groups at P2-cap (e.g., carbamate cap analog **12**) most often led to reduced potency. Aniline-based aryl-oxamides had by far the best potency improvements, where compounds such as **13** (CoV-2 EC_90_ = 45 nM) managed to achieve a >14× improvement in potency over PF-00835231. Aryl-oxamide P2-caps did not pair well with proline P2-derivatives, as exemplified by **15** (CoV-2 EC_90_ = 365 nM) and **16** (CoV-2 EC_90_ = 982 nM).

In addition to the Ames-liability, aryl-oxamides unfortunately also showed general instability in rodent plasma, which was another factor (as rodent toxicology and efficacy studies would have met limiting exposures) in the decision to move away from these potent compounds. We tried to explore isosteric derivatives such as **18** (CoV-2 EC_90_ > 6 µM) or **19** (CoV-2 EC_90_ > 10 µM); significant losses of potency were evident. Removal of one carbonyl motif while retaining a similar count of H-bond donors in **20** (CoV-2 EC_90_ > 9 µM) led to significant activity loss.

Notably, the addition of the extra methyl group at the P2 position of **13** gives a potency advantage of ~3× over **11**, consistent with the hydrophobic nature of the SARS-CoV-2 M^pro^ S2 pocket. This, along with the shift to the six-membered lactam at the P1 sidechain, helped **mCMX110** retain the potency of **8** despite the move away from aryl-oxamides. Moving to a seven-membered lactam P1 in **21** (CoV-2 EC_90_ > 5 µM) did not improve potency. We attempted to incorporate more constrained structural features to mCMX110, as highlighted by bicyclo[1.1.1]butane derivative **22** and cyclobutyl derivative **23**. Such derivatizations failed to provide a superior profile to **mCMX110** regarding the balance between potency, microsomal clearance, and the unbound fraction.

### 3.3. Formulation, Stability, and Solubility Profile of ***mCMX110*** to Enable Oral Administration

**Figure 3:**
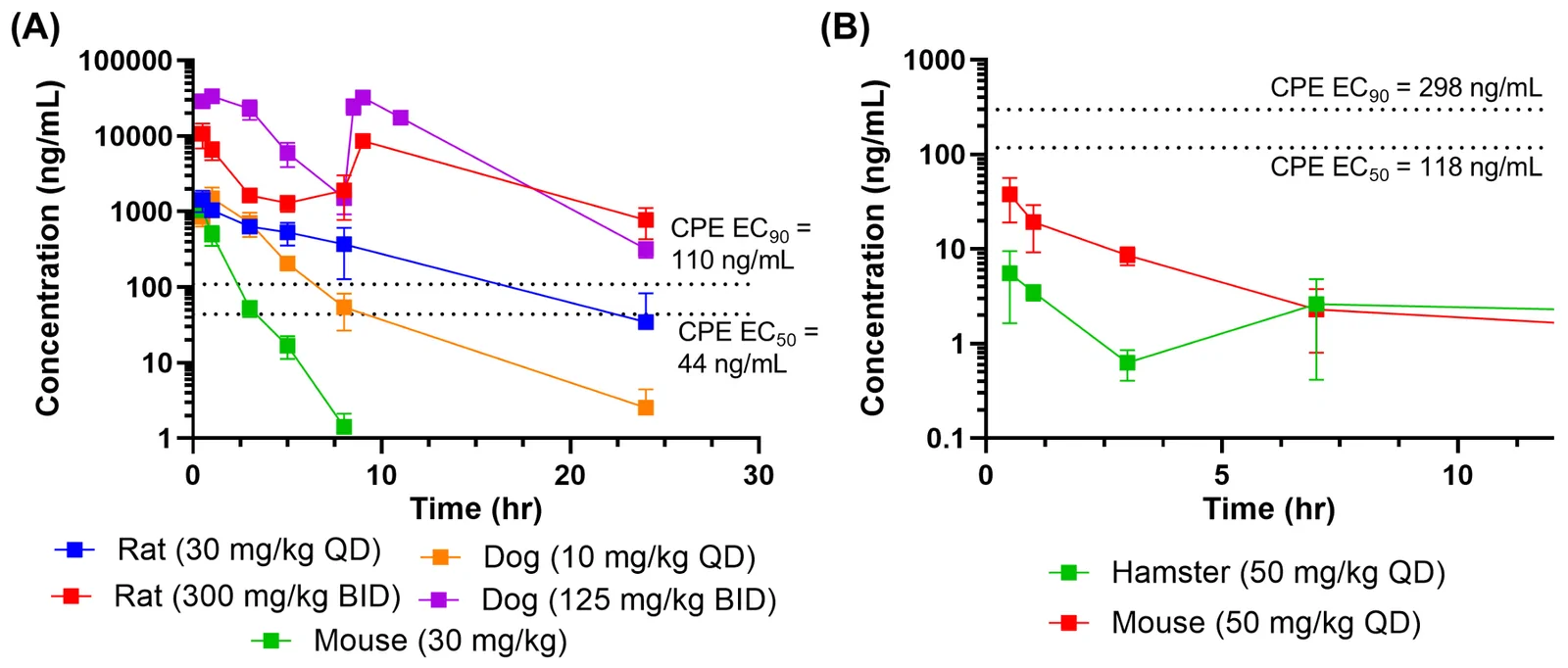
**Oral preclinical pharmacokinetic profile of mCMX110** (**A**) **is superior to PF-00835231** (**B**). **mCMX110** shows superior coverage over SARS-CoV-2 CPE EC_90_ and has good dose-proportionality to exposure in both rats and dogs. Except for where otherwise specified, all represented PK studies were performed in 50% PEG300, 10% ethanol, and 40% saline. **mCMX110** dog 125 mg/kg BID oral dosing performed using a suspension formulation in 0.5% NaCMC (aqueous), and rat 300 mg/kg BID oral dosing performed using a suspension formulation in 0.5% MC (aqueous). Oral bioavailability (F_PO_): PF-00835231 = 0.04% (hamster), 1.1% (mouse); **mCMX110** = 18% (mouse), 10–12% (rat), 44–79% (dog).

In general, most PK studies (Figure 3) described in this manuscript for **mCMX110** were run with the amorphous material (DSC T_g_ onset ~66 °C), obtained post SFC purification. **mCMX110,** however, can be crystallized as a stable single polymorph (DSC melting onset = 86.3 °C; peak temperature = 91.6 °C) using an antisolvent crystallization approach (acetone/pentane).

The bulk stability of this crystallized material is moderate, where **mCMX110** demonstrates good stability under humid conditions at 40 °C over short durations, but undergoes substantial degradation at elevated temperatures (60 °C) and with prolonged exposure. For one tested batch, the initial purity of 97.7% showed minimal change after one week at 40 °C/75% RH (96.8% open; 97.3% closed), while a significant decrease was observed at 60 °C (58.4%). As anticipated, pH-dependent aqueous and biorelevant fluid (FesSIF, FasSIF, SGF) solubility is fairly high (≥2 mg/mL) for **mCMX110**, enabling up to 100 mg/mL homogeneous suspensions in simple aqueous media (f.ex, 0.5% MC or 0.5% NaCMC), which worked well in PK studies (Figure 3, Table 2). Regarding stability in biorelevant media, **mCMX110** was very stable in SIF and SGF, whereas PF-00835231 was found (Table 2) to have poor SIF stability, which may in part contribute to the poor oral bioavailability of this comparator.

### 3.4. Preclinical Oral Pharmacokinetics of ***mCMX110*** Outperforms *PF-00835231*

When given as an oral dose in simple aqueous suspensions, **mCMX110** readily covers its SARS-CoV-2 EC_90_ threshold target (Figure 3—dotted lines represented in respective graphs). In mice, as anticipated, faster clearance makes the coverage less profound at a low dose (30 mg/kg). Still, even in mice, plasma exposure above the EC_90_ threshold for **mCMX110** is improved relative to PF-00835231. In addition, **mCMX110** showed good dose-escalation feasibility in both rats and dogs, resulting in good proportionality to exposure increases. In dog PO PK, **mCMX110** demonstrated consistently high oral bioavailability (44–79%) across multiple studies. Notably, early aryl-oxamide lead **8** had a better performance than PF-00835231 (supporting information) but generally lacked stability in rodent plasma (Table 2). This was another key factor supporting the selection of **mCMX110** as the lead compound, in which the plasma stability issue was solved.

### 3.5. ***mCMX110*** Has a Good Preclinical Safety Profile In Vitro and In Vivo

The in vitro potency profile of **mCMX110** was not driven by any cytotoxicity, and CC_50_ was always above the top concentrations measured in representative assays (>30 µM, Table 2). Oral administration of **mCMX110** in dogs at a 125 mg/kg/dose for 5 days BID did not result in adverse effects, including effects on behavioral, hematologic, renal, or coagulation parameters. Similarly, repeat-dose tolerability study in rats for 5 days (100–300 mg/kg/dose BID PO) did not reveal any prohibitive findings. **mCMX110** had no effect on the inhibition of the five Cyp isoforms tested and was negative for mutagenicity in a mini-Ames assay (Table 2).

## 4. Discussion

Conventionally, hydroxymethyl ketone (HMK) warhead bearing compounds are often perceived as less favorable for oral delivery, due to the presence of an additional hydrogen bond donor in the molecule and the ensuing polarity and potential metabolic liabilities. The present work expands the chemical space of SARS-CoV-2 M^pro^ inhibitors by revisiting this HMK warhead space and demonstrating that their historical limitations can be mitigated through the holistic optimization of molecular properties rather than warhead substitution alone. While recent coronavirus covalent inhibitor clinical success has largely centered on nitrile-based inhibitors, our findings highlight that HMKs remain a viable and differentiated modality with favorable selectivity and tunable pharmacokinetic properties.

In this work, **mCMX110** was identified as a lead compound that achieves a balanced profile across potency and physicochemical properties, and in vivo exposure. Compared to PF-00835231, **mCMX110** demonstrates ~3× improved antiviral potency, both biochemically and in cells. Importantly, these gains were achieved without increasing lipophilicity, as reflected in the maintained or improved ligand efficiency metrics. In addition, **mCMX110** also reduced plasma protein binding, which helps drive a substantially higher unbound fraction in vivo. This improvement in free drug exposure is particularly relevant in the context of antiviral efficacy, where target engagement is governed by unbound concentrations. If combined, the greater unbound exposure/potency may translate into improved antiviral efficacy for this compound in future studies.

Our SAR exploration underscored some key design principles for HMK warhead-based M^pro^ inhibitors. This is important as our extensive work in this area has established that not all warheads pair similarly with different P2/P3 groups. The use of aryl-oxamide motifs as P2-caps led to marked potency gains, consistent with the potential favorable interactions within the S3/S4 pockets [21], resulting in early lead **8**. However, these aryl-oxamides drove mutagenicity signals in Ames assays, and had general plasma instability in rodents. The eventual transition to non-anilinic oxamides in **mCMX110** preserved potency while resolving these liabilities, illustrating the importance of balancing target engagement with developability constraints. Expansion of the P1 lactam from a five- to a six-membered ring contributed to potency improvements, likely by better complementing the S1 subsite geometry. Further expansion to a seven-membered ring (e.g., **21**) resulted in loss of potency.

A distinguishing feature of **mCMX110** is its favorable oral pharmacokinetic profile, achieved without the need for prodrug strategies or pharmacokinetic boosters. Unlike PF-00835231, which suffers from poor oral exposure and instability in simulated intestinal fluid, **mCMX110** exhibits good stability in simulated intestinal and gastric fluids and has oral exposure reaching >50% in higher animals. **mCMX110** also has excellent aqueous solubility (>2 mg/mL) and suspendability, enabling robust formulability as simple aqueous suspensions. These properties subsequently translate into robust in vivo exposure across species, with oral dosing achieving plasma concentrations exceeding the SRAS-CoV-2 antiviral EC_90_ threshold (Figure 3). Although species-related differences in oral-bioavailability (Figure 3 and Table 2) exist, dose-proportional exposure and sustained target coverage were observed in both rodent and non-rodent species, supporting the potential for clinically relevant oral dosing regimens. However, early human dose predictions (not detailed in this disclosure) for **mCMX110** projected a >1 g/day dose to cover the target C_min_ threshold at steady state. Ideally, we would prefer the daily dose burden to be <500 mg in standalone therapy (no ritonavir boosting). Therefore, additional improvements are needed for **mCMX110** prior to translation into the clinic.

From a safety perspective, **mCMX110** demonstrated a clean in vitro profile, with no observable cytotoxicity at concentrations well above antiviral potency and no detectable CYP inhibition across major isoforms. Furthermore, repeat oral dosing in rats and dogs (5 days BID) was well tolerated, with no adverse findings in clinical chemistry or behavioral assessments. Although additional toxicological evaluation is required, these early results are encouraging and consistent with the generally favorable selectivity profile of HMK warheads.

It is our view that further optimization of this scaffold is likely feasible to improve upon the potency and stability properties of **mCMX110**. As immediate next steps, we plan to use **mCMX110** (or an improved analog thereof) in a preclinical efficacy study to establish proof-of-concept efficacy data, and plan to generate co-crystal structure data for **mCMX110** with SARS-CoV-2 M^pro^. In addition to structure-guided further potency optimization of the lead, the improved analogs may also include Lufotrelvir-like prodrug modifications that are hypothesized to enhance the bulk stability and the oral bioavailability of **mCMX110**.

## 5. Conclusions

This work demonstrates that hydroxymethyl ketone warhead-based compounds can be advanced as orally bioavailable antivirals with competitive potency and favorable pharmacological properties. The discovery of **mCMX110** provides a compelling example of how revisiting underexplored warheads can yield differentiated candidates that address the limitations of current therapies. More broadly, these findings reinforce the importance of maintaining chemical diversity in antiviral drug discovery to ensure preparedness against current and future coronavirus threats.

## Figures and Tables

**Table 1:** Representative compounds and profiling data for HMK series SAR.

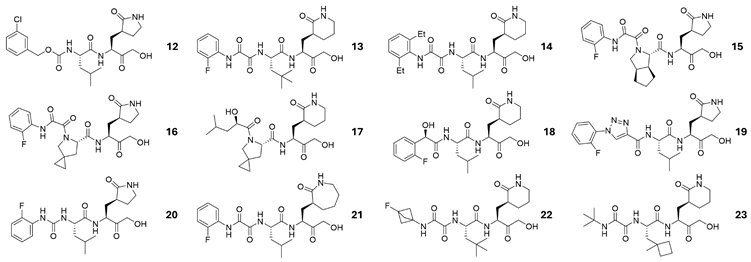						
Compound	clogP	CoV-2 IC_50_^a^ (nM)	CoV-2 Potency		hPPB (%)	HLM Cl_int_ (µL/min/mg)
			EC_90_ (nM) ^b^	LLE_EC_90_		
**8**	−0.13	160	245 ± 109 (21)	6.7	51.8	13.0
**9**	−0.61	n.d.	680 ± 117 (3)	6.8	n.d.	7.2
**10**	1.28	n.d.	993 (1)	4.7	n.d.	n.d.
**11**	0.26	44	121 ± 48 (31)	6.7	44.6	28.5
**12**	1.55	n.d.	1584 ± 84 (2)	4.2	n.d.	89.4
**13**	0.65	20	45 ± 4 (6)	6.7	64.5	50.0
**14**	1.24	19	59 (1)	6.0	n.d.	117
**15**	−0.04	28	365 ± 62 (6)	6.5	51.5	14.1
**16**	−0.28	n.d.	982 ± 171 (2)	6.3	n.d.	n.d.
**17**	−0.26	82	>5 µM (2)	5.1	16.7	2.4
**18**	0.35	n.d.	>5 µM (3)	4.9	n.d.	n.d.
**19**	0.12	166	>5 µM (1)	4.9	44.3	23.0
**20**	0.93	n.d.	>5 µM (1)	4.1	n.d.	n.d.
**21**	0.65	138	>5 µM (2)	4.6	55.3	31.3
**22**	−0.37	34	317 ± 80 (2)	6.9	29.1	25.6
**23**	−0.07	n.d.	183 ± 26 (2)	6.8	39.0	58.4
**mCMX110**	−0.22	35	241 ± 74 (34)	6.8	17.8	29.5
PF-00835231	0.89	101	630 ± 310 (69)	5.3	77.2	13.9

EC_90_ = 90% effective concentration as measured in HeLa-ACE2 cells; LLE = lipophilic ligand efficiency as calculated from EC_90_ = pEC_90_–clogP; Cl_int_ = intrinsic clearance;; HLM = human liver microsomes; hPPB = human plasma protein binding; n.d. = not determined. ^a^ IC_50_ values are from an individual experiment; ^b^ cell-based EC_90_ values are the arithmetic average of multiple independent experiments (n), each with technical triplicates per experiment. Geometric means for EC_90_ of mCMX110 = 232 nM and PF-00835231 = 586 nM.

**Table 2:** Comparative performance of HMK compounds **8** and **mCMX110** vs. PF-00835231.

	PF-00835231	8	mCMX110
MW [g/mol]	472.5	464.5	454.6
**In Vitro Potency**			
SARS-CoV-2 M^pro^ IC_50_ [nM]	101	160	35
HeLa ACE2 CoV-2 EC_50/90_ [nM]	250/630	118/245	97/241
CoV-1/MERS-CoV EC_50_ [nM]	n.d.	n.d.	13/16
HeLa ACE2 CoV-2 EC_50/90_ [ng/mL]	118/298	55/114	44/110
LLE_CPE_EC_90,CoV-2_	5.3	6.7	6.8
hPPB adjusted CoV-2 EC_90_ (ng/mL)	1304	236	134
HCT-8 hCoV-OC43 EC_90_ [µM]	3.48	2.61	5.63
MRC5 hCoV-229E EC_90_ [nM]	963	291	930
HeLa ACE2 CoV-2 *variants* EC_90_ [nM] alpha/omicron	1720/759	n.d.	339/255
Uninfected Cytotoxicity CC_50_ [μM] HeLa-ACE2/HCT-8/MRC5	>40/>30/>30	>40/>30/>30	>40/>30/>30
**ADME Properties**			
Microsomal/hepatocyte stability [CL_int_, µL/min/mg; CL_hep_, µL/min/10^6^]	13.9/1.8 (human)	13.0/1.8 (human)	29.5/0.8 (human)
SIF/SGF stability [% at 2 h]	9/98	100/100	96/95
PPB [%] M/R/D/C/Hu	84.4/91.0/81.7/78.9/77.2	U/U/38.5/48.7/51.8	63.1/89.8/34.5/15.0/17.8
P_app(a->b/b->a)_ Caco-2 [10^−6^ cm/s]	BQL/1.74	0.2/6.9	BQL/2.9
Aqueous Solubility [pH 7.4, mg/mL]	<0.1 [12]	n.d.	>2
**In Vivo Properties**			
Plasma clearance [IV PK CLp; mL/min/kg]	86.4 (M)/18.4 (Ha)	U (M)/23.3 (D)	83.7 (M)/15.3 (R)/17.2 (D)/32.6 (C)
IV PK Vss [L/kg]	1.9 (Vd, M)/0.50 (Vd Ha)	U (M)/1.0 (D)	0.78 (M)/1.3 (Vd, R)/0.93 (D)/1.5 (C)
F_PO_ (%)	1.1% (M)/0.04% (Ha)	<1% (M)/5–8% (Ha)/38% (D)	18% (M)/10–12% (R)/44–79% (D)
**Mutagenicity and DDI potential**			
CYP inhibition IC_50_ (1A2, 2C19, 2C9, 2D6, 3A4)	n.d.	>50 µM (all)	>50 µM (all)
Ames assay	n.d.	n.d.	Negative

Unless otherwise specified, all PK performed with solutions containing 50% PEG 300, 10% ethanol, and 40% saline. Abbreviations: M = mice; Ha = hamster; R = rat; D = dog; C = cyno; Hu = human; U = unstable; n.d. = not done; BQL = below quantifiable level.

## Data Availability

The original contributions presented in this study are included in the article/Supplementary Material. Further inquiries can be directed to the corresponding author.

## Supplementary Materials

The following supporting information can be downloaded at: https://www.mdpi.com/article/10.3390/v18080821/s1.

## Abbreviations

The following abbreviations are used in this manuscript:

ADME	Absorption, Distribution, Metabolism, and Excretion
CPE	Cytopathic effect
DSC	Differential Scanning Calorimetry
FesSIF	Fed-state simulated intestinal fluid
FasSIF	Fasted-state simulated intestinal fluid
HLM	Human liver microsomes
HMK	Hydroxymethyl ketone
IV	Intravenous
LLE	Lipophilic ligand efficiency
MC	Methylcellulose
M^pro^	Main protease
NaCMC	Sodium carboxymethyl cellulose
NMR	Nuclear magnetic resonance
PK	Pharmacokinetics
PO	Oral administration
PPB	Plasma protein binding
RH	Relative humidity
SAR	Structure–Activity Relationship
SARS-CoV	Severe acute respiratory syndrome coronavirus
SFC	Supercritical fluid chromatography
SGF	Simulated gastric fluid
SIF	Simulated intestinal fluid
Vd	Volume of distribution
Vss	Volume of distribution at steady state
WH	Warhead
