# Supplementary material for: Orally Bioavailable SARS-CoV-2 Protease Inhibitors Bearing a Hydroxymethyl Ketone Warhead
Source: Viruses. 2026 Jul 26;18(8):821. doi: 10.3390/v18080821 (PMC13517159; doi:10.3390/v18080821)

Supporting Information

for

Orally Bioavailable SARS-CoV-2 Protease Inhibitors Bearing a Hydroxymethyl Ketone Warhead

N. G. R. Dayan Elshan[†,*], Karen C. Wolff[†], Frank Weiss[†], Sourav Ghorai[†], Gennadii Grabovyi[†], Katy Wilson[†], Laura Riva[†], Ashley K. Woods[†], James Pedroarena[†], Armen Nazarian[†], Yuyin Liu[†], Wrickban Mazumdar[†], Lirui Song[†], Neechi Okwor[†], Jacqueline Malvin[†], Malina A. Bakowski[†], Melanie G. Kirkpatrick[†], Amal Gebara-Lamb[†], Edward Huang[†], Vân T. B. Nguyen-Tran[†], Stuart Weston[¥], Carly Dillen[¥], Victor Chi[†], Shuangwei Li[†], Matthew Frieman[¥], Sumit K. Chanda[†], Kyoung-Jin Lee[†], Case W. McNamara[†], Anil Kumar Gupta[†], Alireza Rahimi[†], Jian Jeffrey Chen[†], Sean B. Joseph[†], Peter G. Schultz[†,‡], Arnab K. Chatterjee[†]

[†]Calibr-Skaggs Institute for Innovative Medicines, The Scripps Research Institute, La Jolla, CA 92037, USA.

[‡]Department of Chemistry, The Scripps Research Institute, La Jolla, CA 92037, USA.

[¥]Department of Microbiology and Immunology, University of Maryland School of Medicine, Baltimore, MD 21201, USA.

[*]Corresponding author

Contents:

Description	Page Number
1H NMR Spectra for Compounds **8-23**	S2
**mCMX110**: Spectral Data	S10
Oral PK Profile of Compound **8** in Hamster and Dog	S12

^{1}H NMR Spectra for Compounds 8-23

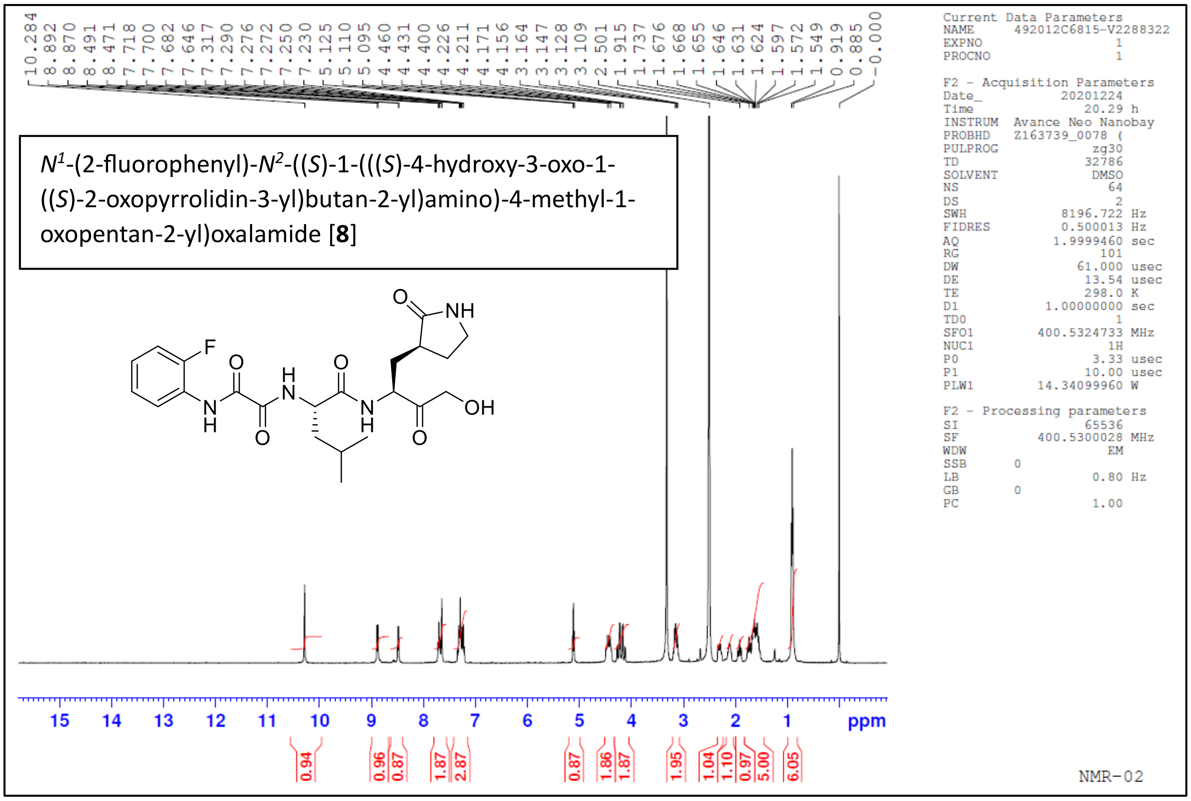

N^1-(2-fluorophenyl)-N^2-((*S*)-1-(((*S*)-4-hydroxy-3-oxo-1-((*S*)-2-oxopyrrolidin-3-yl)butan-2-yl)amino)-4-methyl-1-oxopentan-2-yl)oxalamide [**8**]

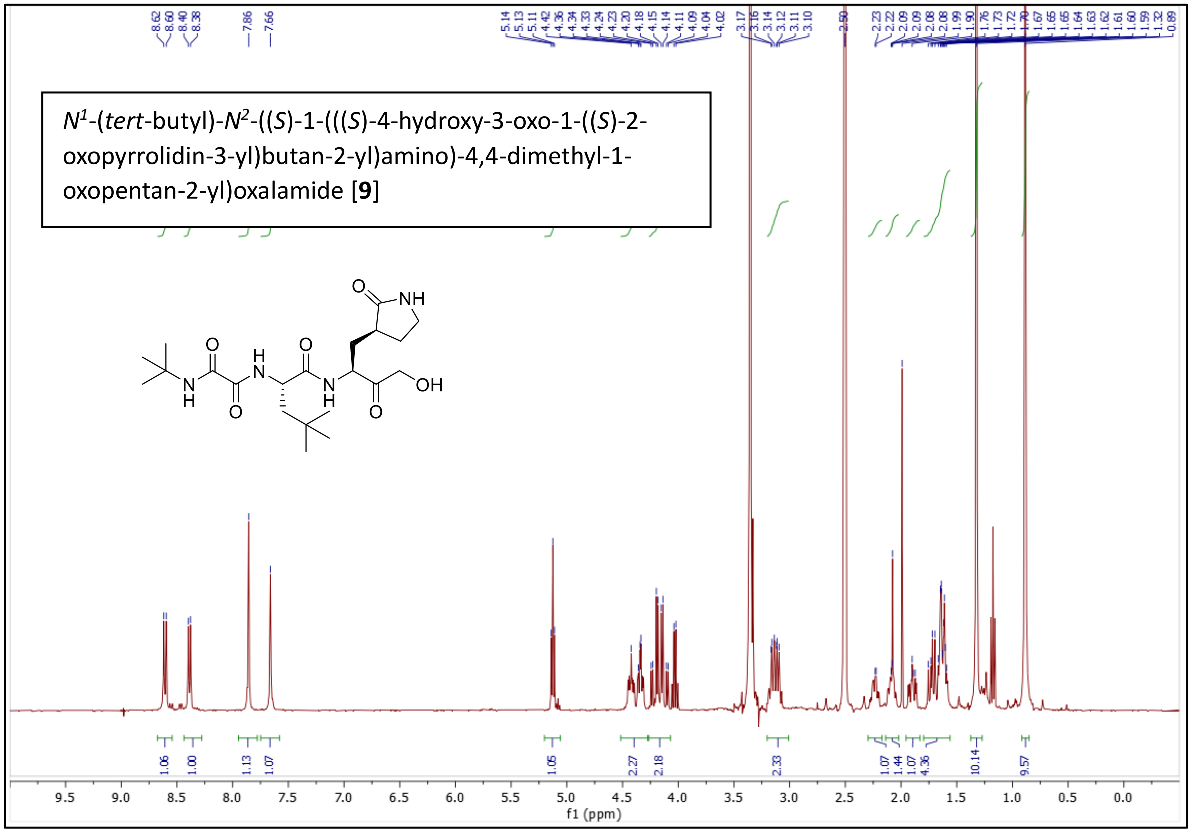

N^1-(*tert*-butyl)-N^2-((*S*)-1-(((*S*)-4-hydroxy-3-oxo-1-((*S*)-2-oxopyrrolidin-3-yl)butan-2-yl)amino)-4,4-dimethyl-1-oxopentan-2-yl)oxalamide [**9**]

N-((*S*)-1-(((*S*)-4-hydroxy-3-oxo-1-((*S*)-2-oxopiperidin-3-yl)butan-2-yl)amino)-4-methyl-1-oxopentan-2-yl)-4-methoxy-1H-indole-2-carboxamide [**10**]

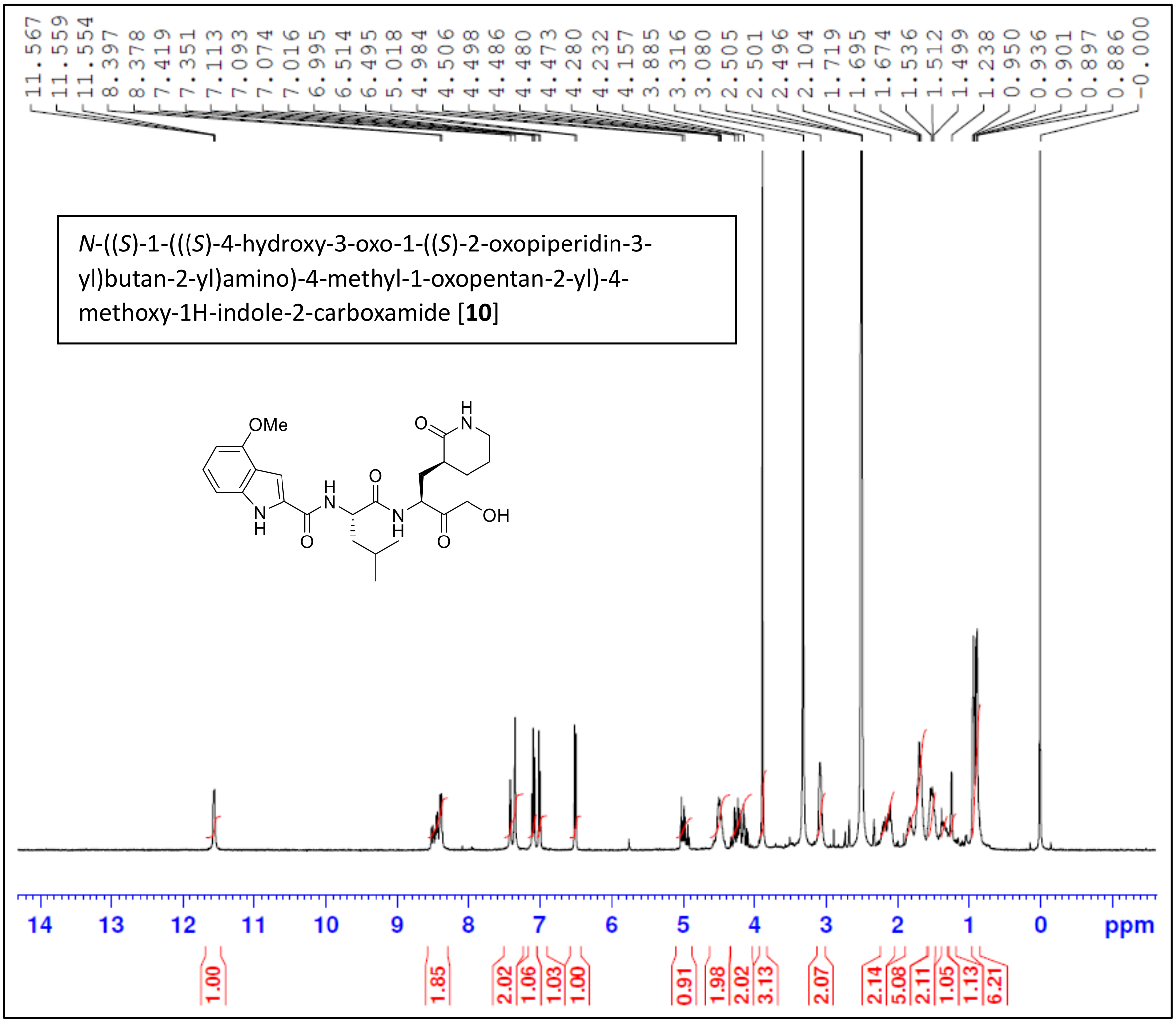

N[1]-(2-fluorophenyl)-*N2*-((*S*)-1-(((*S*)-4-hydroxy-3-oxo-1-((*S*)-2-oxopiperidin-3-yl)butan-2-yl)amino)-4-methyl-1-oxopentan-2-yl)oxalamide [**11**]

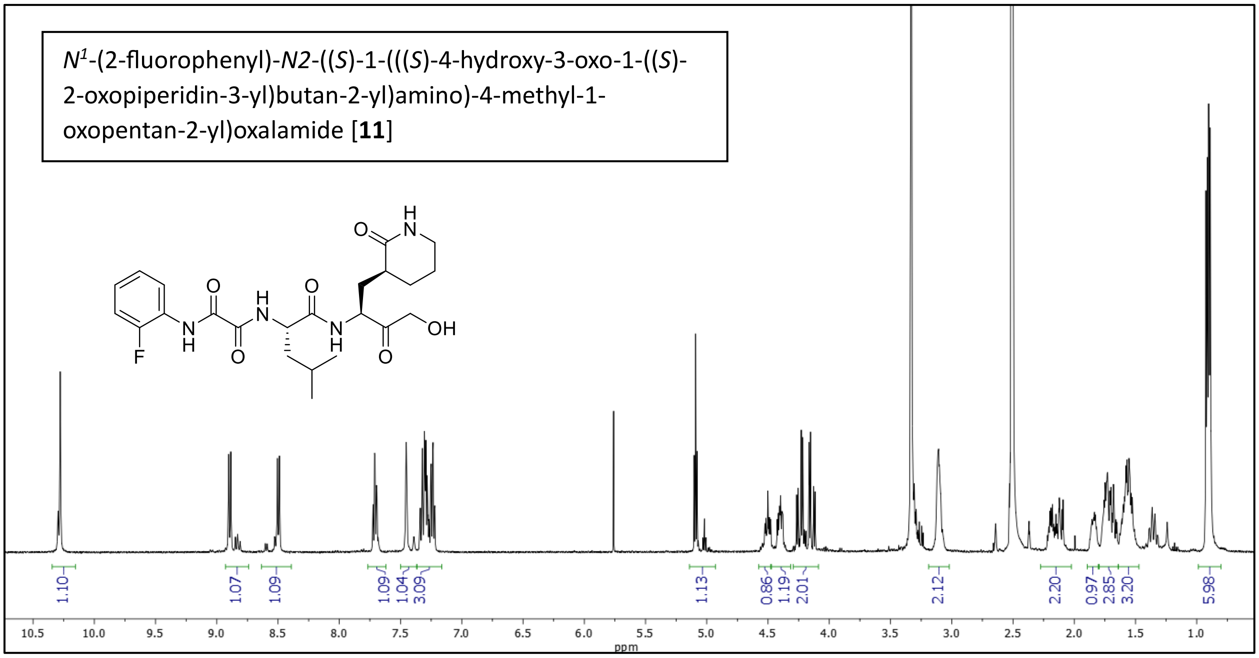

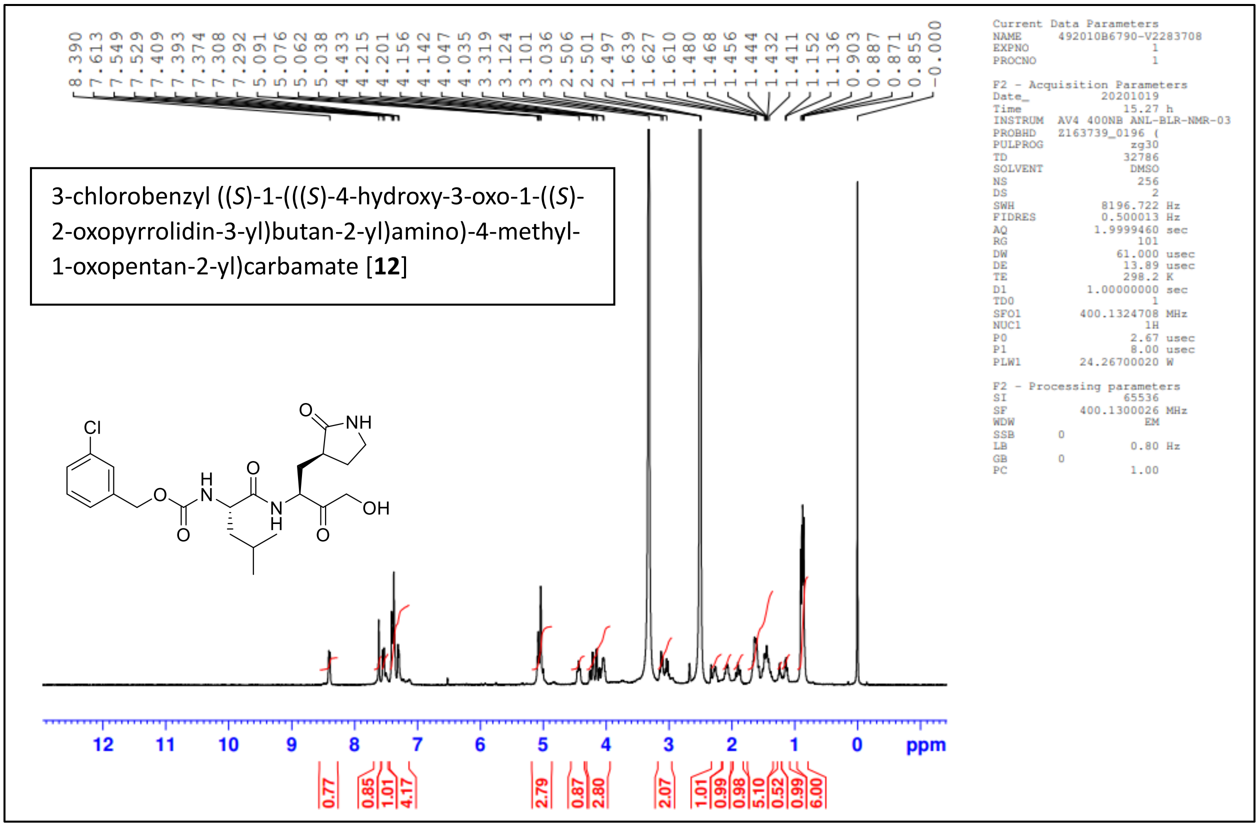
3-chlorobenzyl ((*S*)-1-(((*S*)-4-hydroxy-3-oxo-1-((*S*)-2-oxopyrrolidin-3-yl)butan-2-yl)amino)-4-methyl-1-oxopentan-2-yl)carbamate [**12**]
8.390 7.613 7.549 7.529 7.409 7.393 7.374 7.308 7.292 5.091 5.076 5.062 5.038 4.433 4.215 4.201 4.156 4.142 4.047 4.035 3.319 3.124 3.101 3.036 2.506 2.501 2.497 1.639 1.627 1.610 1.480 1.468 1.456 1.444 1.432 1.411 1.152 1.136 0.903 0.887 0.871 0.855 -0.000
Current Data Parameters
NAME 492010B6790-V2283708
EXPNO 1
PROCNO 1
F2 - Acquisition Parameters
Date_ 20201019
Time 15.27 h
INSTRUM AV4 400NB ANL-BLR-NMR-03
PROBHD Z163739_0196 (
PULPROG zg30
TD 32786
SOLVENT DMSO
NS 256
DS 2
SWH 8196.722 Hz
FIDRES 0.500013 Hz
AQ 1.9999460 sec
RG 101
DW 61.000 usec
DE 13.89 usec
TE 298.2 K
D1 1.00000000 sec
TD0 1
SFO1 400.1324708 MHz
NUC1 1H
P0 2.67 usec
P1 8.00 usec
PLW1 24.26700020 W
F2 - Processing parameters
SI 65536
SF 400.1300026 MHz
WDW EM
SSB 0
LB 0.80 Hz
GB 0
PC 1.00
12 11 10 9 8 7 6 5 4 3 2 1 0 ppm
0.77 0.85 1.01 4.17 2.79 0.87 2.80 2.07 1.01 0.99 0.98 5.10 0.52 0.99 6.00

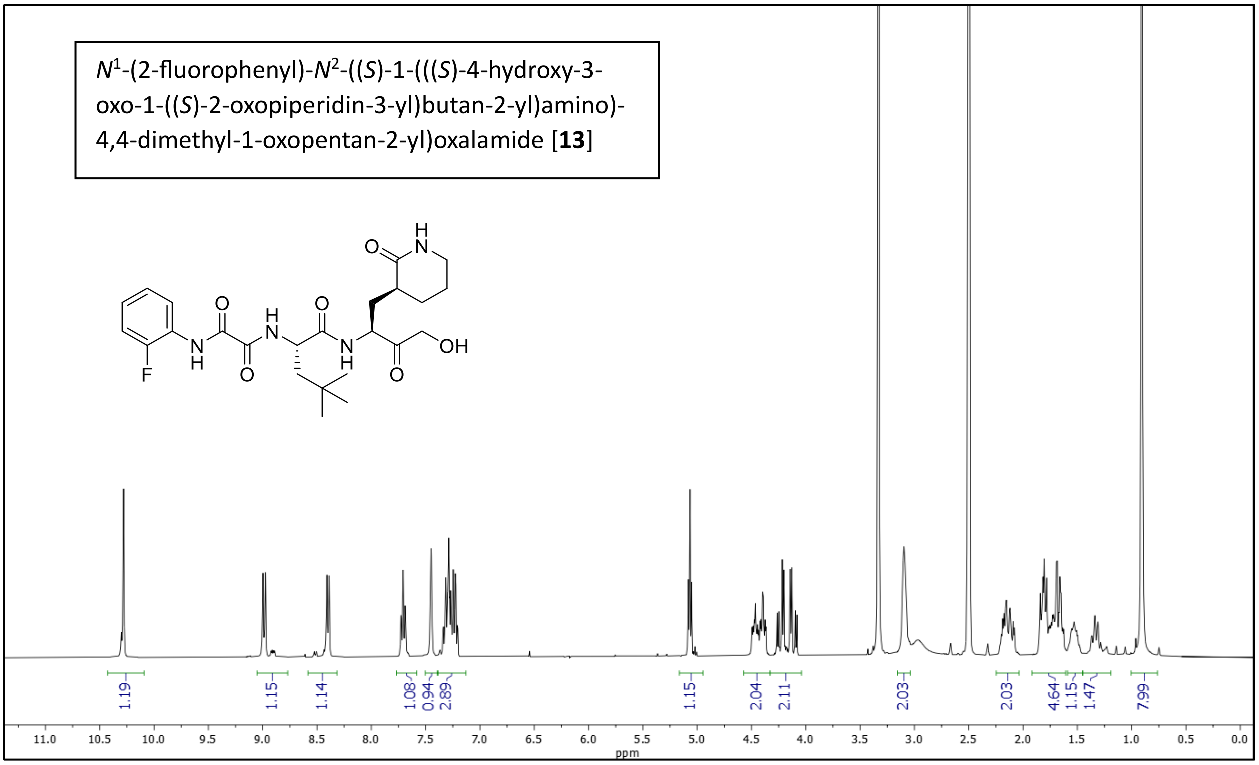
N[1]-(2-fluorophenyl)-*N*[2]-((*S*)-1-(((*S*)-4-hydroxy-3-oxo-1-((*S*)-2-oxopiperidin-3-yl)butan-2-yl)amino)-4,4-dimethyl-1-oxopentan-2-yl)oxalamide [**13**]
1.19 1.15 1.14 1.08 0.94 2.89 1.15 2.04 2.11 2.03 2.03 4.64 1.15 1.47 7.99
11.0 10.5 10.0 9.5 9.0 8.5 8.0 7.5 7.0 6.5 6.0 5.5 5.0 4.5 4.0 3.5 3.0 2.5 2.0 1.5 1.0 0.5 0.0
ppm

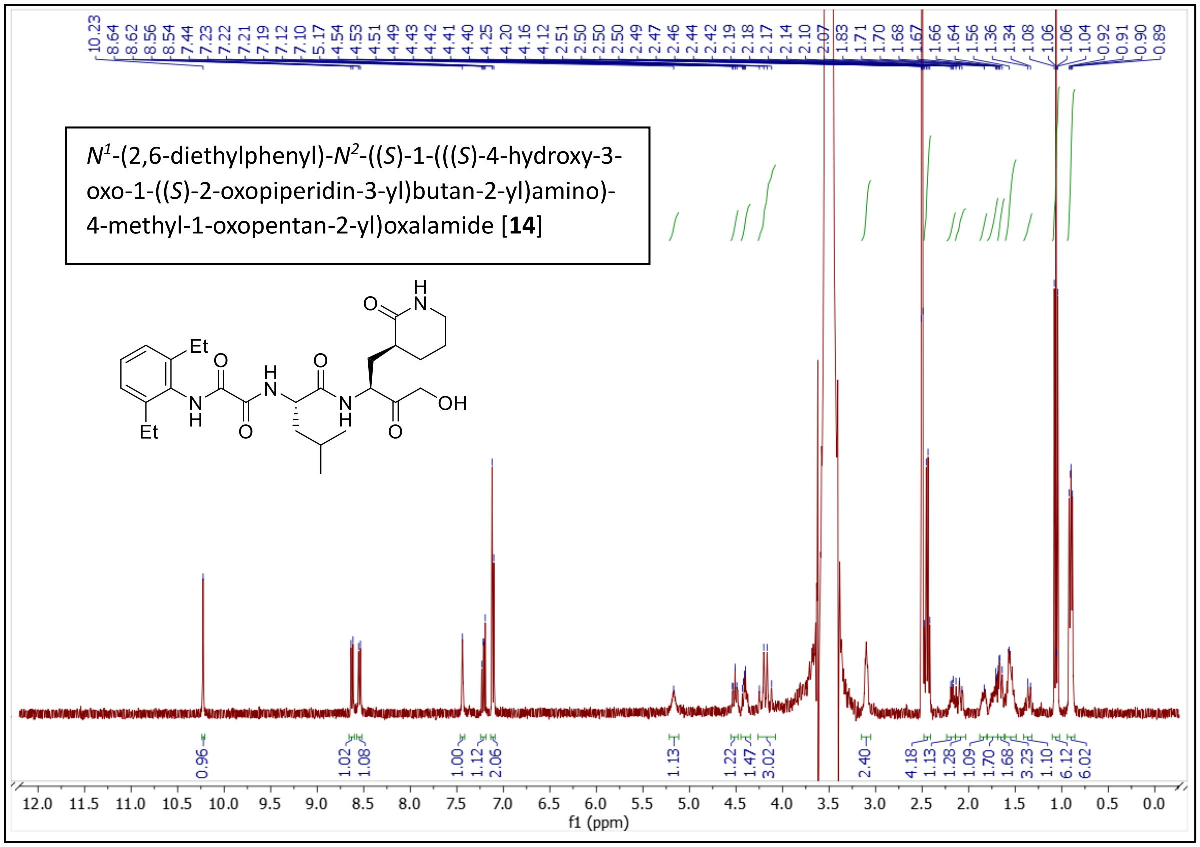
10.23
8.64
8.62
8.56
8.54
7.44
7.23
7.22
7.21
7.19
7.12
7.10
5.17
4.54
4.53
4.51
4.49
4.43
4.42
4.41
4.40
4.25
4.20
4.16
4.12
2.51
2.50
2.50
2.50
2.49
2.47
2.46
2.44
2.42
2.19
2.18
2.17
2.14
2.10
2.07
1.83
1.71
1.70
1.68
1.67
1.66
1.64
1.56
1.36
1.34
1.08
1.06
1.06
1.04
0.92
0.91
0.90
0.89
N[1]-(2,6-diethylphenyl)-*N*[2]-((*S*)-1-(((*S*)-4-hydroxy-3-oxo-1-((*S*)-2-oxopiperidin-3-yl)butan-2-yl)amino)-4-methyl-1-oxopentan-2-yl)oxalamide [**14**]
0.96
1.02
1.08
1.00
1.12
2.06
1.13
1.22
1.47
3.02
2.40
4.18
1.13
1.28
1.09
1.70
1.68
3.23
1.10
6.12
6.02
12.0 11.5 11.0 10.5 10.0 9.5 9.0 8.5 8.0 7.5 7.0 6.5 6.0 5.5 5.0 4.5 4.0 3.5 3.0 2.5 2.0 1.5 1.0 0.5 0.0
f1 (ppm)

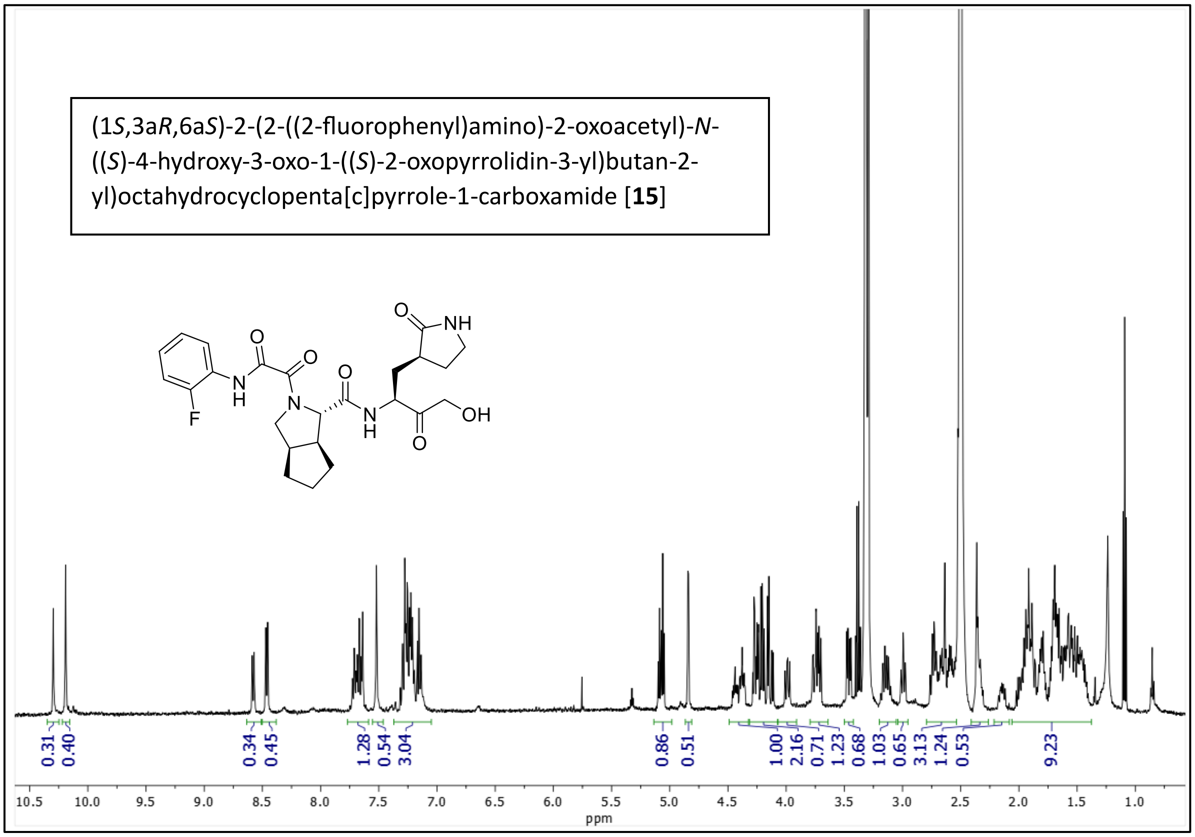
(1*S*,3a*R*,6a*S*)-2-(2-((2-fluorophenyl)amino)-2-oxoacetyl)-*N*-((*S*)-4-hydroxy-3-oxo-1-((*S*)-2-oxopyrrolidin-3-yl)butan-2-yl)octahydrocyclopenta[c]pyrrole-1-carboxamide [**15**]
0.31
0.40
0.34
0.45
1.28
0.54
3.04
0.86
0.51
1.00
2.16
0.71
1.23
0.68
1.03
0.65
3.13
1.24
0.53
9.23
10.5 10.0 9.5 9.0 8.5 8.0 7.5 7.0 6.5 6.0 5.5 5.0 4.5 4.0 3.5 3.0 2.5 2.0 1.5 1.0
ppm

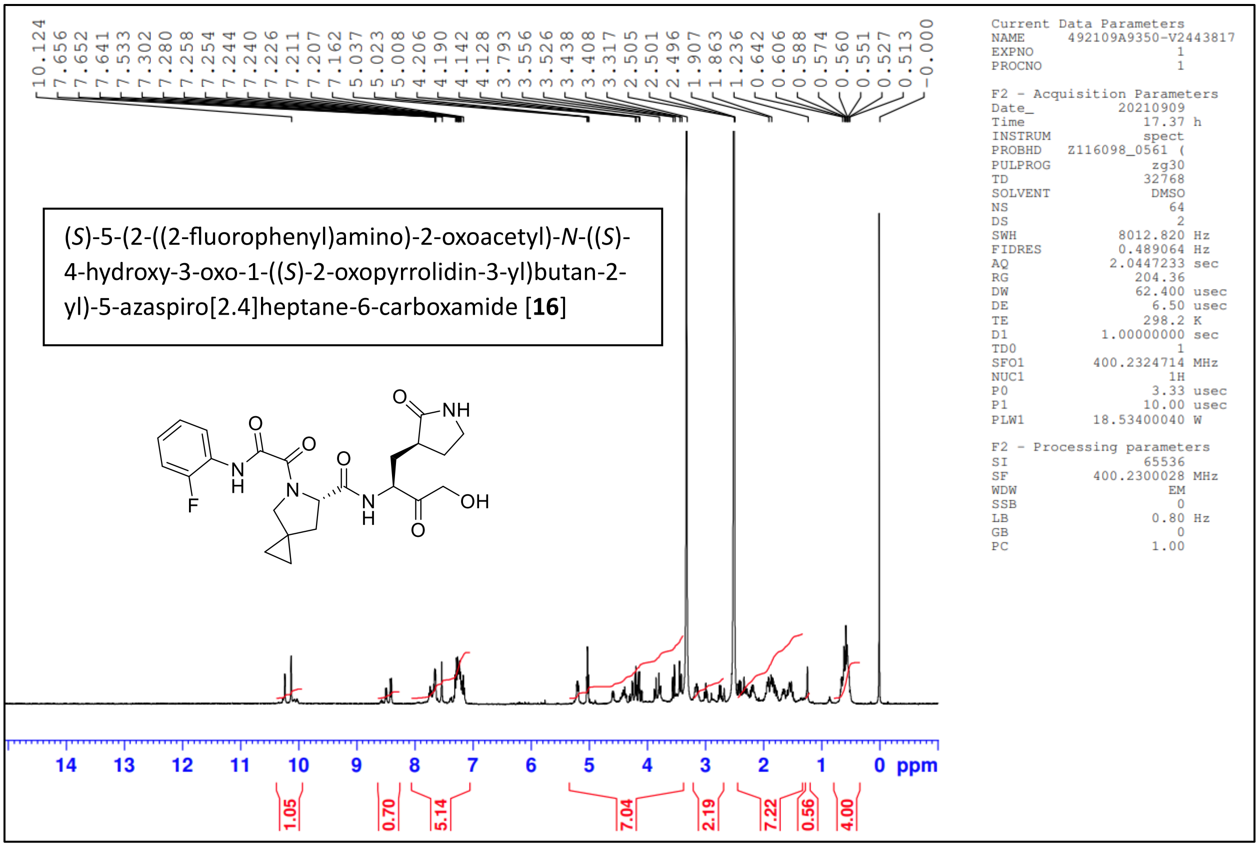

(*S*)-5-(2-((2-fluorophenyl)amino)-2-oxoacetyl)-*N*-((*S*)-4-hydroxy-3-oxo-1-((*S*)-2-oxopyrrolidin-3-yl)butan-2-yl)-5-azaspiro[2.4]heptane-6-carboxamide [**16**]

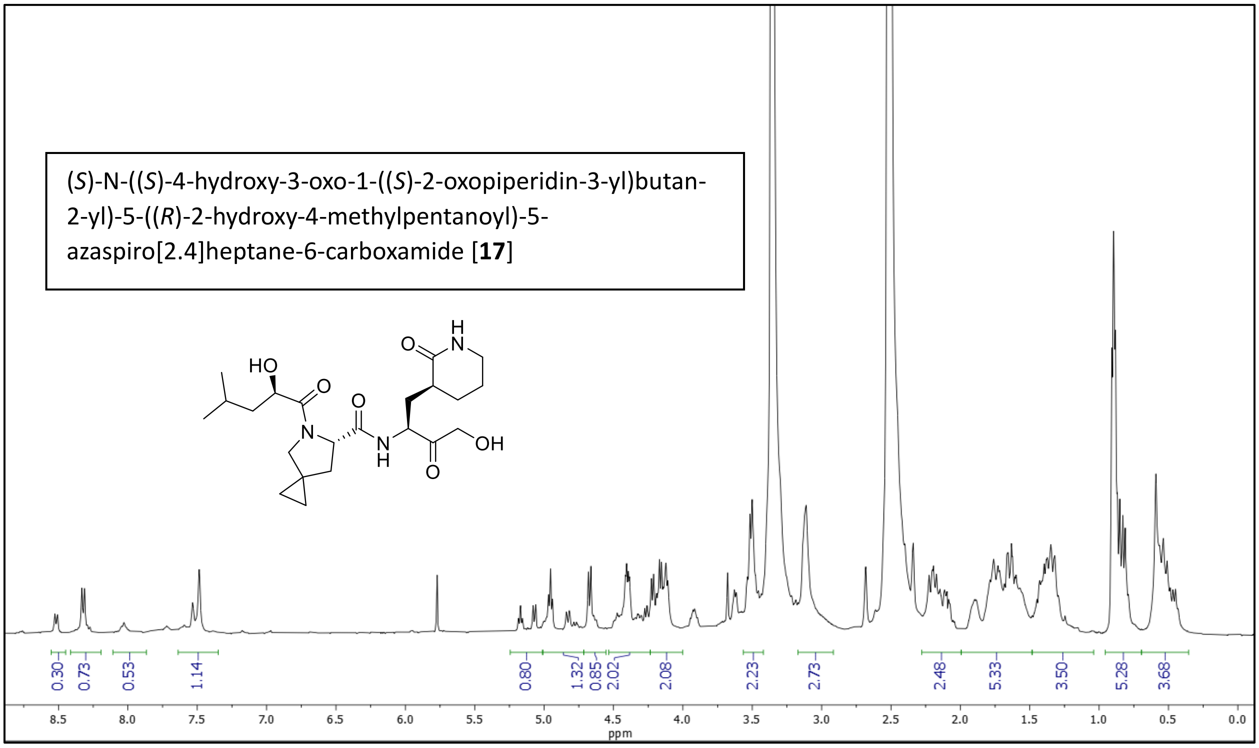

(*S*)-N-((*S*)-4-hydroxy-3-oxo-1-((*S*)-2-oxopiperidin-3-yl)butan-2-yl)-5-((*R*)-2-hydroxy-4-methylpentanoyl)-5-azaspiro[2.4]heptane-6-carboxamide [**17**]

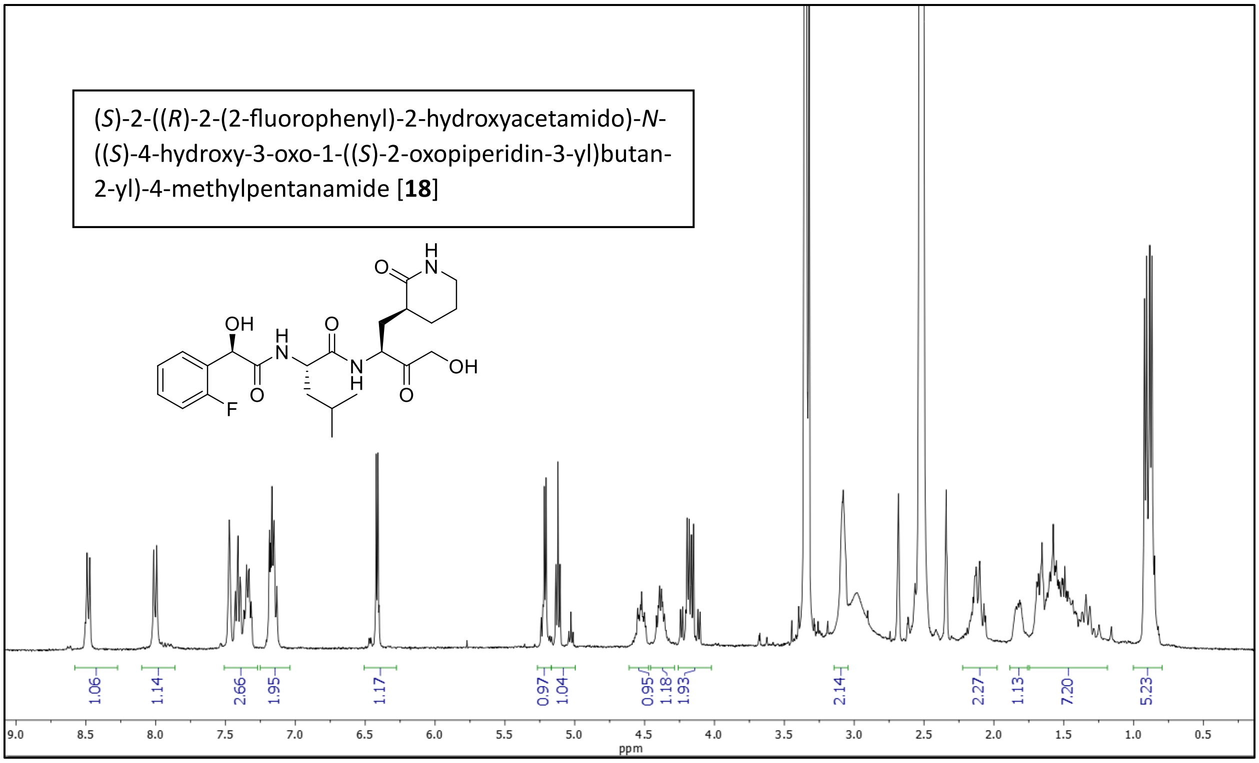
(S)-2-((R)-2-(2-fluorophenyl)-2-hydroxyacetamido)-N-((S)-4-hydroxy-3-oxo-1-((S)-2-oxopiperidin-3-yl)butan-2-yl)-4-methylpentanamide [18]
ppm

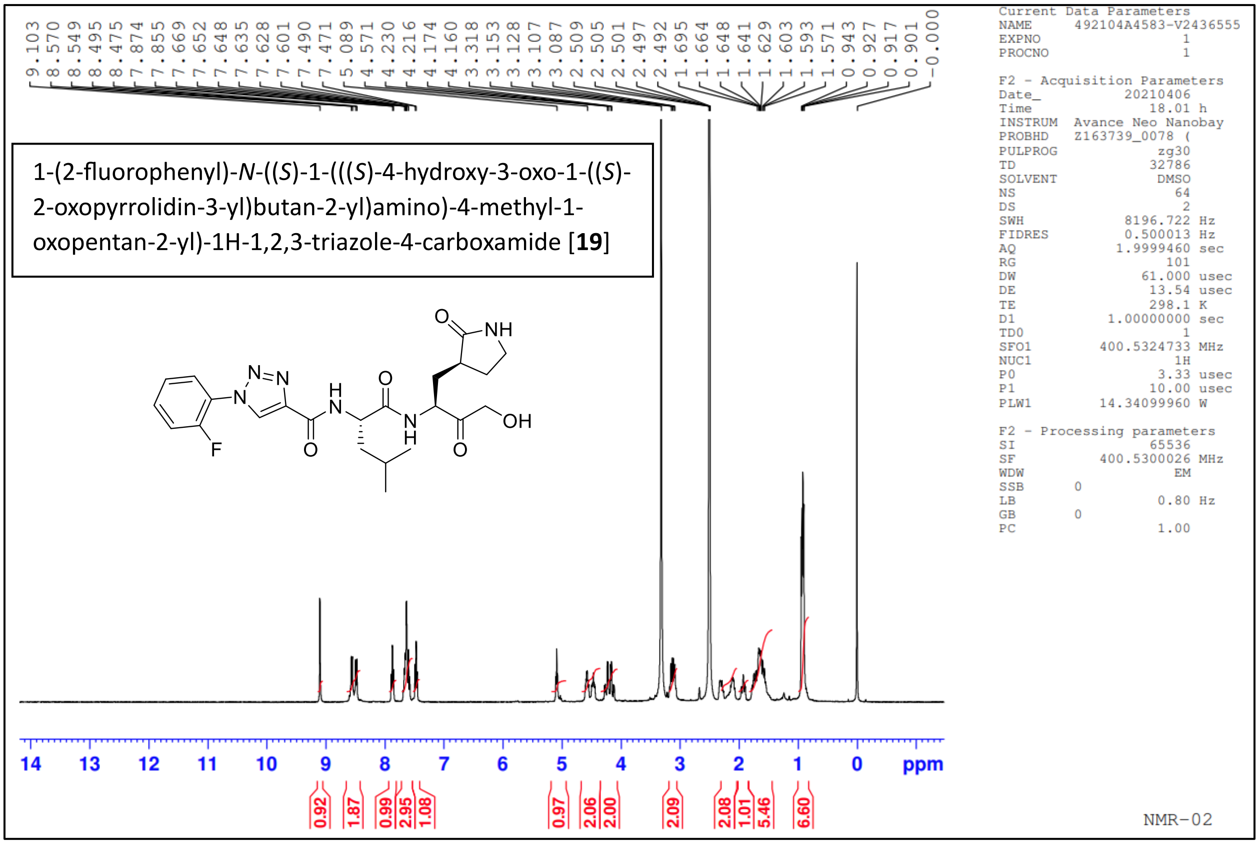
1-(2-fluorophenyl)-N-((S)-1-(((S)-4-hydroxy-3-oxo-1-((S)-2-oxopyrrolidin-3-yl)butan-2-yl)amino)-4-methyl-1-oxopentan-2-yl)-1H-1,2,3-triazole-4-carboxamide [19]
Current Data Parameters
NAME 492104A4583-V2436555
EXPNO 1
PROCNO 1
F2 - Acquisition Parameters
Date_ 20210406
Time 18.01 h
INSTRUM Avance Neo Nanobay
PROBHD Z163739_0078 (
PULPROG zg30
TD 32786
SOLVENT DMSO
NS 64
DS 2
SWH 8196.722 Hz
FIDRES 0.500013 Hz
AQ 1.9999460 sec
RG 101
DW 61.000 usec
DE 13.54 usec
TE 298.1 K
D1 1.00000000 sec
TD0 1
SFO1 400.5324733 MHz
NUC1 1H
P0 3.33 usec
P1 10.00 usec
PLW1 14.34099960 W
F2 - Processing parameters
SI 65536
SF 400.5300026 MHz
WDW EM
SSB 0
LB 0.80 Hz
GB 0
PC 1.00
ppm
NMR-02

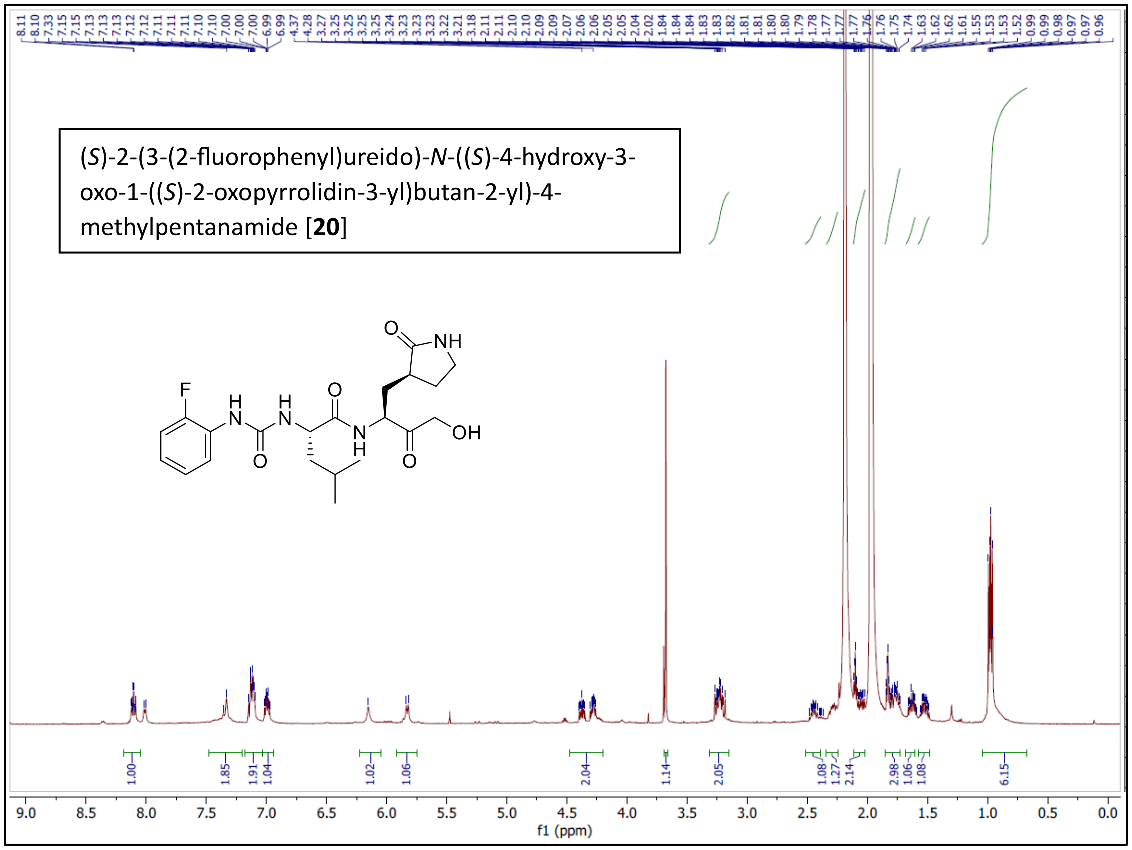
(S)-2-(3-(2-fluorophenyl)ureido)-N-((S)-4-hydroxy-3-oxo-1-((S)-2-oxopyrrolidin-3-yl)butan-2-yl)-4-methylpentanamide [20]
f1 (ppm)

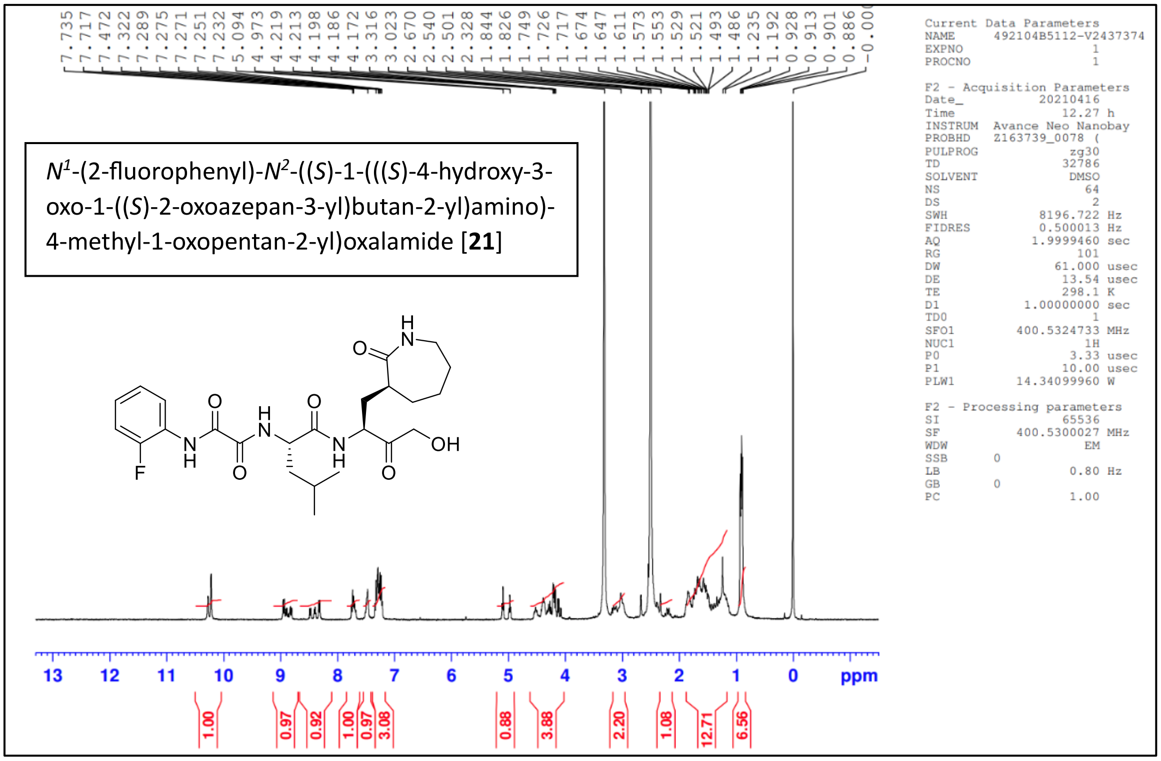
N1-(2-fluorophenyl)-N2-((S)-1-(((S)-4-hydroxy-3-oxo-1-((S)-2-oxoazepan-3-yl)butan-2-yl)amino)-4-methyl-1-oxopentan-2-yl)oxalamide [21]
Current Data Parameters
NAME 492104B5112-V2437374
EXPNO 1
PROCNO 1
F2 - Acquisition Parameters
Date_ 20210416
Time 12.27 h
INSTRUM Avance Neo Nanobay
PROBHD Z163739_0078 (
PULPROG zg30
TD 32786
SOLVENT DMSO
NS 64
DS 2
SWH 8196.722 Hz
FIDRES 0.500013 Hz
AQ 1.9999460 sec
RG 101
DW 61.000 usec
DE 13.54 usec
TE 298.1 K
D1 1.00000000 sec
TD0 1
SFO1 400.5324733 MHz
NUC1 1H
P0 3.33 usec
P1 10.00 usec
PLW1 14.34099960 W
F2 - Processing parameters
SI 65536
SF 400.5300027 MHz
WDW EM
SSB 0
LB 0.80 Hz
GB 0
PC 1.00
ppm

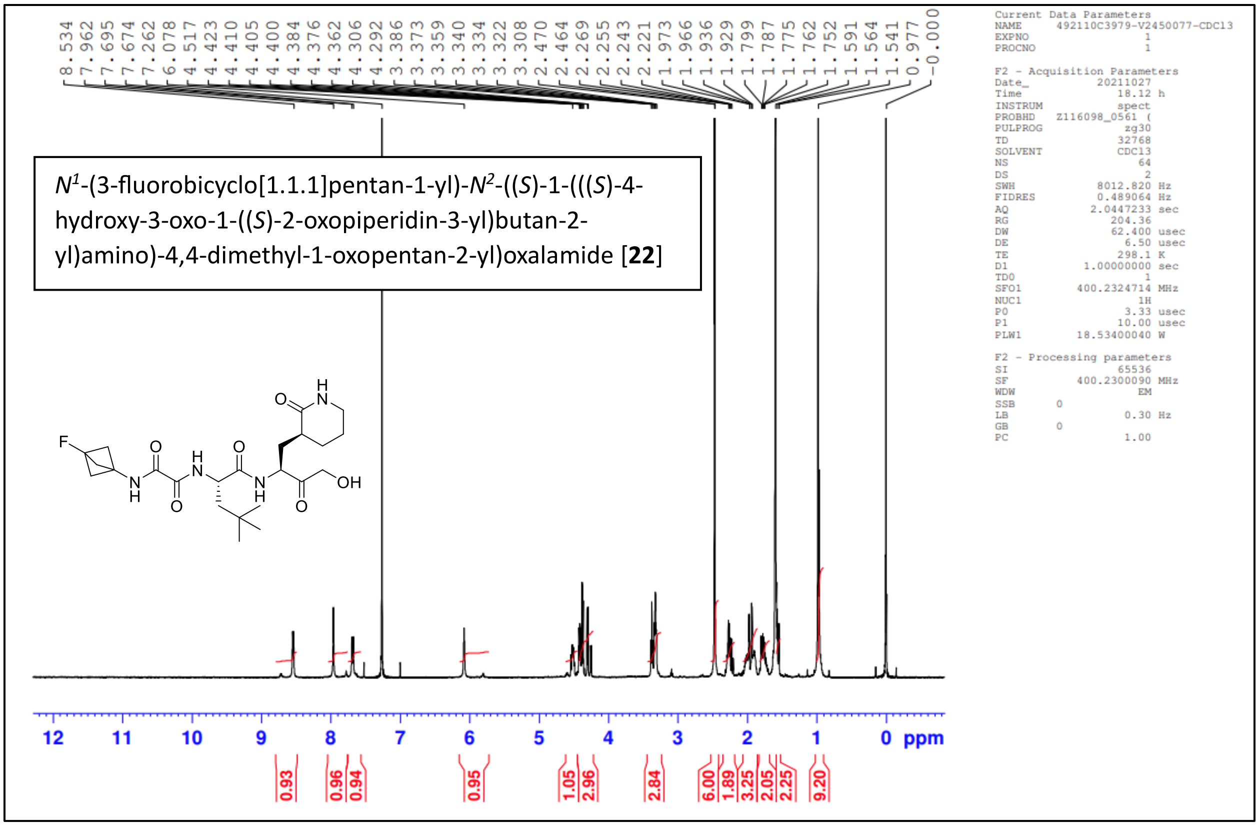

N[1]-(3-fluorobicyclo[1.1.1]pentan-1-yl)-*N*[2]-((*S*)-1-(((*S*)-4-hydroxy-3-oxo-1-((*S*)-2-oxopiperidin-3-yl)butan-2-yl)amino)-4,4-dimethyl-1-oxopentan-2-yl)oxalamide [**22**]

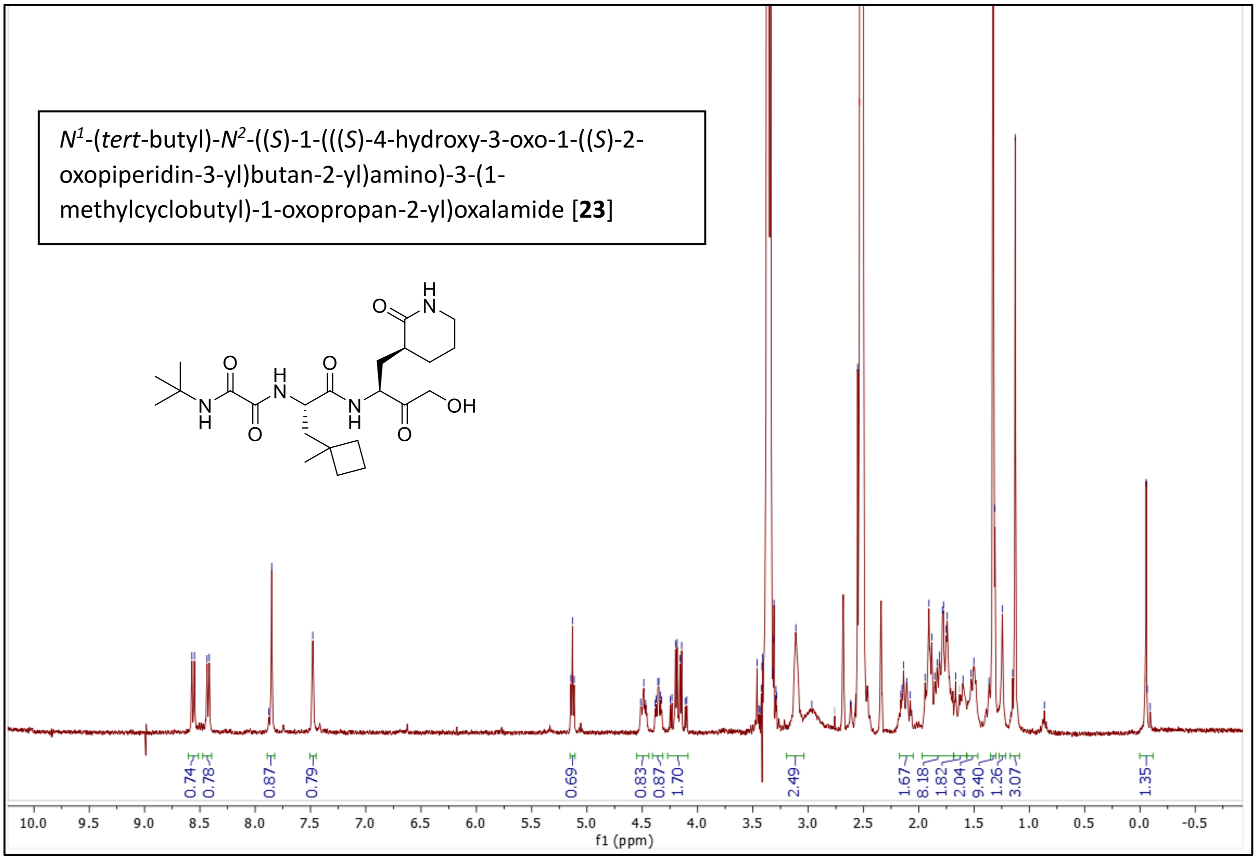

N[1]-(*tert*-butyl)-*N*[2]-((*S*)-1-(((*S*)-4-hydroxy-3-oxo-1-((*S*)-2-oxopiperidin-3-yl)butan-2-yl)amino)-3-(1-methylcyclobutyl)-1-oxopropan-2-yl)oxalamide [**23**]

mCMX110: Spectral Data

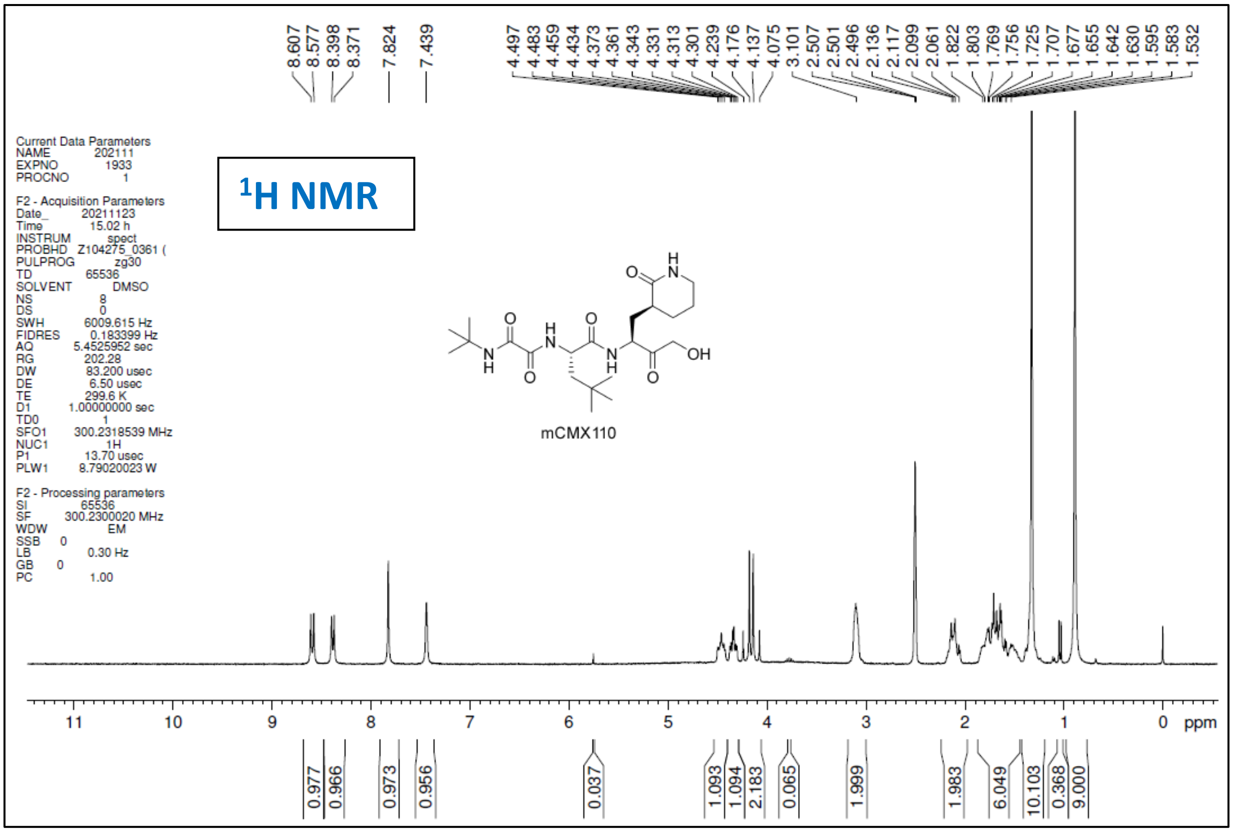

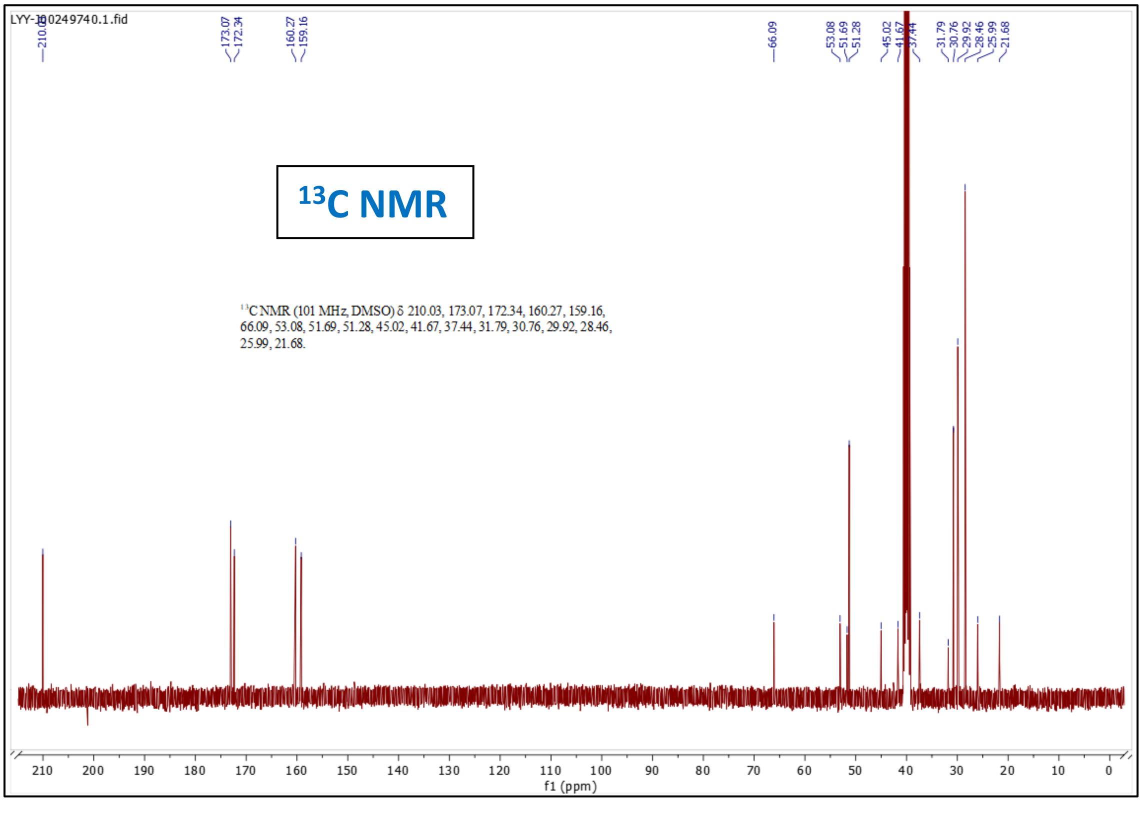

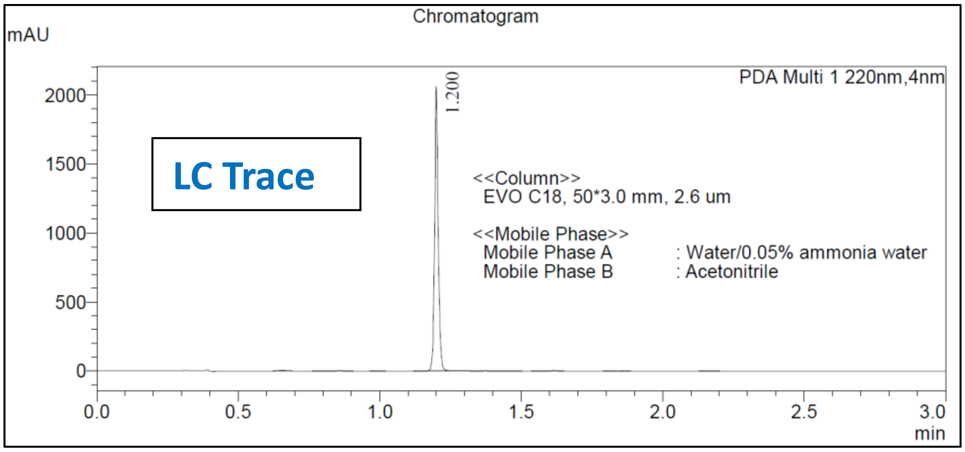
Chromatogram
mAU
PDA Multi 1 220nm,4nm
LC Trace
1.200
<<Column>>
EVO C18, 50*3.0 mm, 2.6 um
<<Mobile Phase>>
Mobile Phase A : Water/0.05% ammonia water
Mobile Phase B : Acetonitrile
2000
1500
1000
500
0
0.0
0.5
1.0
1.5
2.0
2.5
3.0
min

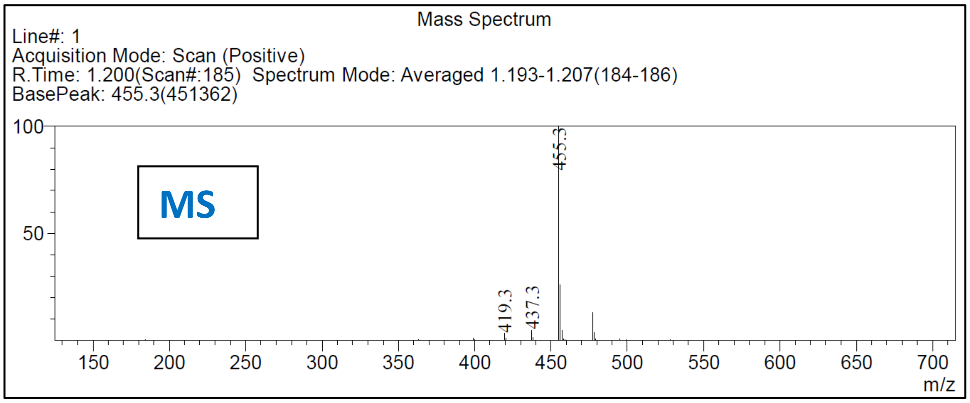
Mass Spectrum
Line#: 1
Acquisition Mode: Scan (Positive)
R.Time: 1.200(Scan#:185) Spectrum Mode: Averaged 1.193-1.207(184-186)
BasePeak: 455.3(451362)
MS
455.3
419.3
437.3
100
50
150
200
250
300
350
400
450
500
550
600
650
700
m/z

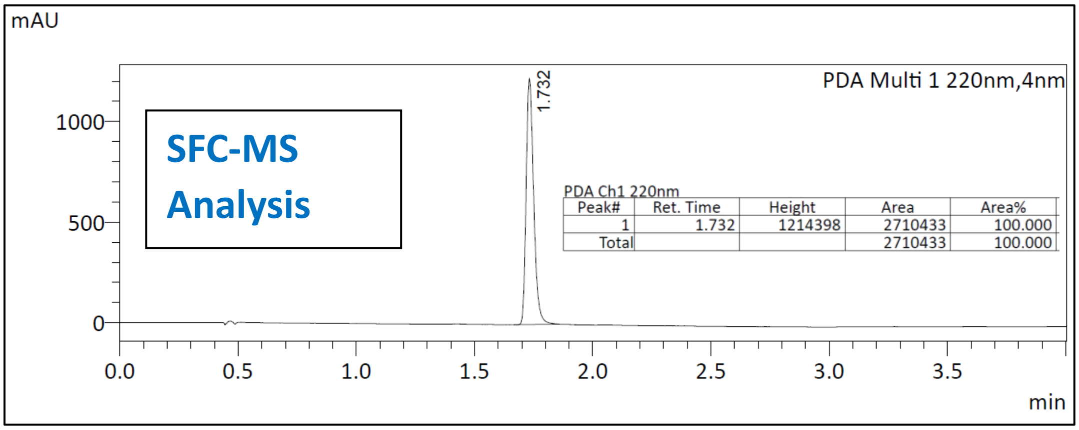
mAU
PDA Multi 1 220nm,4nm
SFC-MS Analysis
1.732
PDA Ch1 220nm
Peak# Ret. Time Height Area Area%
1 1.732 1214398 2710433 100.000
Total 2710433 100.000
1000
500
0
0.0
0.5
1.0
1.5
2.0
2.5
3.0
3.5
min

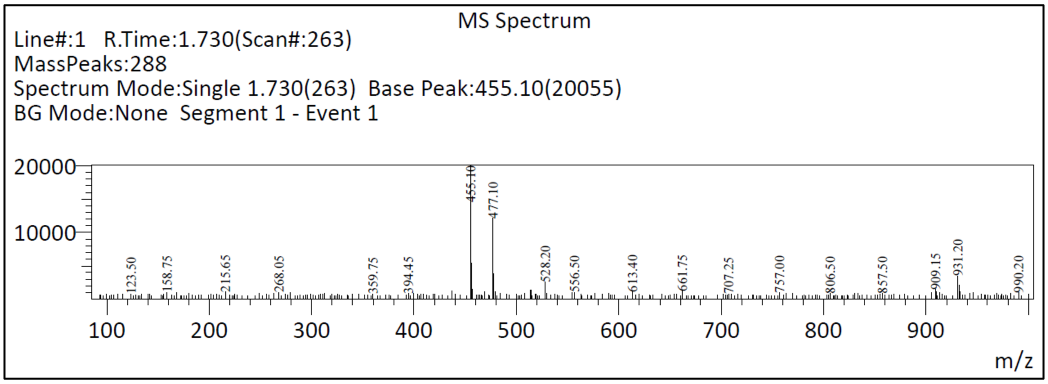
MS Spectrum
Line#:1 R.Time:1.730(Scan#:263)
MassPeaks:288
Spectrum Mode:Single 1.730(263) Base Peak:455.10(20055)
BG Mode:None Segment 1 - Event 1
20000
10000
455.10
477.10
123.50
158.75
215.65
268.05
359.75
394.45
528.20
556.50
613.40
661.75
707.25
757.00
806.50
857.50
909.15
931.20
990.20
100
200
300
400
500
600
700
800
900
m/z

Oral PK Profile of 8 in Hamster and Dog

- Compound was unstable in mouse and rodent plasma, necessitating evaluation in hamsters to obtain measurable PK exposure in rodents.
- Dosing formulation for all studies = 50% PEG 300, 10% ethanol, and 40% saline.
- Dose Normalized IV AUC = 158 hr.ng/mL; Hamster F_{PO} = 5-8%; Dog F_{PO} = 38%

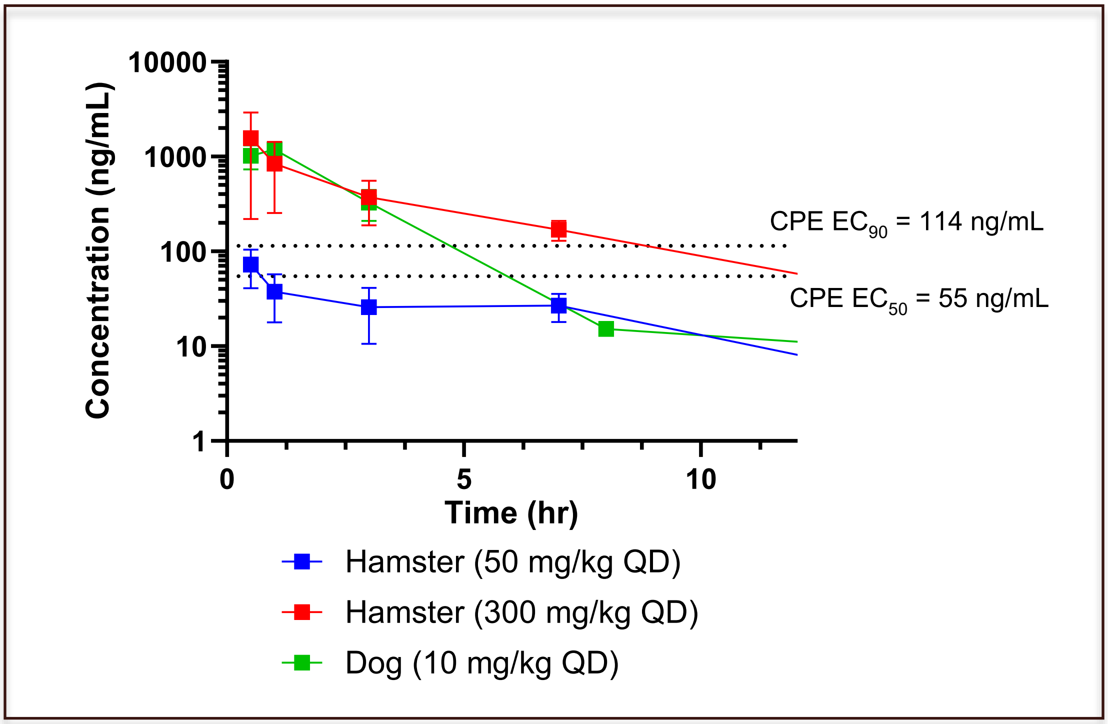

Supplement: Supplementary file 1 [file viruses-18-00821-s001.zip › viruses-4360926-supplementary.pdf]
